# Medical mistrust among diverse survivors of sexual violence: implications for primary health care delivery and engagement

**DOI:** 10.1186/s12875-025-03105-4

**Published:** 2025-12-15

**Authors:** Katherine M. Anderson, Kiyomi Tsuyuki, Alexandra Fernandez DeSoto, Jamila K. Stockman

**Affiliations:** 1 Department of Behavioral, Social, and Health Education Sciences, Emory University Rollins School of Public Health, 1518 Clifton Rd N E, Atlanta, GA 30322 USA; 2 Division of Infectious Diseases and Global Public Health, Department of Medicine, University of California San Diego School of Medicine, 9500 Gilman Dr, La Jolla, 92093 CA USA; 3 Department of Dermatology, University of California San Diego School of Medicine, 9500 Gilman Dr, La Jolla, 92093 CA USA

**Keywords:** Medical Mistrust, Sexual Violence, Health Care Seeking, Discrimination, Support, Suspicion, Rape, Sexual Assault, Cisgender Women

## Abstract

**Background:**

In the United States, approximately 25% of women experience attempted/ completed rape, and substantially more experience sexual assault, with minoritized women disproportionately. Survivors of sexual violence (SV) face higher odds of poor physical or mental health, leading to frequent interactions with healthcare systems. At the same time, survivors of SV may avoid care due to fear of re-traumatization, structural barriers, economic barriers, or fear and stigma. Despite high rates of medical mistrust (MM) among minoritized groups and previously identified associations between MM and health care avoidance, MM among SV survivors in this context has not been widely explored.

**Methods:**

The THRIVE Study was a prospective case-control study of cisgender female past-month survivors of forced/threatened vaginal sex (rape) compared to consensually sexually active controls. Women completed three visits over three months, inclusive of surveys and biological sample collection. At baseline, participants were asked about past experiences of SV, including “sexual assault,” past “forced/threatened sex” (not inclusive of past-month experiences), and recent “forced/threatened sex” (past month, assessed as eligibility criteria). Binary indicator variables were created for each experience. Using the Group-Based Medical Mistrust scale and sub-scales (discrimination, suspicion, lack of support), differences in MM were examined by SV history and self-identified race and ethnicity.

**Results:**

Survivors of past or recent rape had significantly higher MM than others, with survivors of recent rape having the highest mean MM score (*µ* = 31.00, SD: 9.06 vs. *µ = 25.89*, SD: 9.41, *p* = 0.027; *µ* = 32.28, SD: 8.97 vs. *µ =* 25.86, SD: 9.15, *p* = 0.006). Survivors of past rape had significantly higher *discrimination* domain scores (*p* = 0.027), while recent survivors had significantly higher *suspicion* (*p* = 0.002) and *lack of support* scores (*p* = 0.039). Variation by SV experience and MM domain was identified among Latina, White, and Multiracial women. Black women had significantly higher MM than non-Black women (*µ* = 33.94, SD: 9.39 vs. *µ* = 26.35, SD: 9.00, *p* = 0.005), but no associations with SV history were identified.

**Conclusions:**

Findings underscore the need for (a) patient-centered and trauma-informed care, with implementation of trainings or interventions to increase and knowledge to provide trauma-informed care, and (b) consistent, acceptable screening for experiences of violence across care settings, to inform provider interactions and responses.

**Supplementary Information:**

The online version contains supplementary material available at 10.1186/s12875-025-03105-4.

## Background

In North America, 15% of women and girls over the age of 14 are estimated to have experienced sexual violence (SV) by a non-partner- being forced, coerced or threatened to perform any unwanted sexual act, including rape, attempted rape, unwanted sexual touching or noncontact forms of SV- marking the second highest regional estimate for non-partner SV globally [1]. Additionally, 25% of women in North America have experienced sexual or physical intimate partner violence (IPV) by a former or current partner [2], yielding an even higher estimate for the total burden of SV among women in the United States (U.S.) and Canada. This includes 25% of women in the U.S. who have experienced attempted or completed rape, and over 50% who have experienced some form of contact SV [3]. Lifetime rates of SV are unequally distributed across racial and ethnic groups- almost half of non-Hispanic multiracial women, approximately 44% of non-Hispanic American Indian/Alaskan Native (AI/AN) women, and almost 30% of non-Hispanic White and non-Hispanic Black women are estimated to experience attempted or completed rape, while two-thirds of non-Hispanic multiracial women and 58% of non-Hispanic AI/AN women are estimated to experience unwanted sexual contact [3]. These rates represent disproportionately high rates of SV victimization compared to the general U.S. population.

Survivors of SV often face immediate injury or illness, such as vaginal lacerations, bruises, or sexually transmitted infections (STIs) [4], which may require health care system interactions in the immediate or short-term after violence. In a survey of individuals contacting the National Domestic Violence Hotline, over 60% indicated current health needs associated with their violence experiences [5]. However, survivors of SV also face higher odds of a variety of other adverse health outcomes extending for months to years after violence, including chronic pain and poor physical and/or mental health [4]. Resultingly, survivors of trauma interact with health care systems more frequently than those without trauma [6]. Yet, many survivors of violence choose not to seek care in the aftermath, including medical care. In one national sample of survivors of rape, less than 40% of women sought medical care, with White women, women with income less than $20,000, and women who had experienced forcible rape having higher odds of seeking medical services [7]. A separate national sample found that only 21% of women received medical attention following rape, and 27% of those with injury from rape; in this sample, Black women had significantly higher odds of seeking medical care, as did women with STI, HIV, or pregnancy concerns [8]. Of female and male survivors of rape who sought medical care, less than 30% returned for medical or counseling follow-up after their initial visit [9]; similarly, in a sample of women who sought emergency care following sexual assault, fewer than two in five reported seeing a primary care provider, and of those who did, 25% did not disclose their SV experience to their provider [10].

A variety of reasons have been explored for this lack of service-seeking following SV. Survivors of violence may avoid care due to fear of re-traumatization- a re-experiencing of traumatic stress due to situational stimuli reminiscent of a traumatic experience- which may be of particular concern in the context of physical exams [6]. Women experiencing violence may also face interpersonal or structural barriers to care. Of individuals who utilized the National Domestic Violence Hotline and were contacted for follow-up one or two years later, approximately half indicated that their partner had controlled or restricted access to their health care; others reported financial barriers (53%) and health insurance coverage (38%) as primary barriers to seeking care [5]. Social stigma and fear have likewise been identified as contributors [11].

Medical mistrust (MM) has repeatedly been identified as a prevalent experience among minoritized women, with deleterious impacts for health care seeking [12–14]. Brenick et al. identified that among a sample of Black women who have sex with women, race-based MM was associated with significantly lower engagement in health care [15], while in a random sample of adults in Baltimore, Maryland, MM was significantly positively associated with failure to take medical advice and failure to attend a follow-up appointment [16]. Research has lightly touched on the role of MM in IPV services, particularly as it relates to HIV prevention [17–19]. However, little literature addresses the possible contribution of MM to health care avoidance among survivors of SV. In a 2021 review, Zinzow and colleagues identified “cultural mistrust” as a potential barrier to care-seeking in the aftermath of SV, yet stopped short of specifying mistrust of medical institutions or providers [20]. Importantly, health care relating to sexual and reproductive health, including SV, may be of particular concern to women given historical sexual and reproductive medical abuse. This includes nonconsensual gynecological and reproductive surgeries and experimentation, the Tuskegee Untreated Syphilis Study, compulsory or targeted sterilizations, abortions, and hysterectomies among minoritized women in the name of eugenics [21].

Primary care services can serve an important role in care provision for survivors of SV, both acutely and longitudinally. In addition to providing an opportunity to seek services without disclosure of violence, as might be the case for an emergency room visit, primary care providers often play an essential role in connections to social or mental health services or referrals to specialized care. Yet, the extant literature supports that MM may act as a barrier to seeking primary care services, which may be compounded by experiences of violence. Therefore, the aim of the current analysis is to test differences in MM, overall and subconstructs, by behaviorally- and self-defined SV survivorship status- including recent experiences of forced sex and lifetime experiences of forced sex and sexual assault- across and within racial and ethnic subgroups of participants.

## Methods

The current analysis is a secondary analysis of data from a longitudinal case-control study of women recent survivors of rape and sexually active controls (“The THRIVE Study”). Details of the study have been published in depth elsewhere [22]. Briefly, eligible women experienced forced vaginal penetration by a male in the past month (“cases”) or consensual vaginal sex with a male and no forced vaginal penetration by a male in the past month (“controls”). Enrolled participants completed three study visits (baseline, 1-month, and 3-month), each including a comprehensive interviewer-administered survey, venipuncture blood sample for HIV and inflammatory biomarker testing, and vaginal example including swabs for STIs and collection of cervical-vaginal lavage fluid for testing of genital tract immune biomarkers. Participants also collected saliva samples for three days following each visit, for assessment of stress response dysregulation. Stringent safety protocols were utilized with all participants [22], and research activities were informed by trauma-informed research methods, details of which can be found elsewhere [23]. The current analysis utilizes quantitative survey data from the baseline study visit only.

### Measures

Demographic information was collected from all participants, including gender assigned at birth and gender identity, sexual orientation, age, race and ethnicity, student status, employment status, annual income, and relationship status. The full baseline survey is available in Supplemental File 1.

Typologies of SV are often classified by researchers based on behaviorally-specific categories, yet definitions of these categories vary widely based on researcher assumptions about qualities of violence, such as use of force, intention to cause harm, and definitions of consent [24, 25]. While behaviorally-specific definitions are vital for measures of SV prevalence [26], the words used in these measures or labels researchers give to experiences based on these measures may not align with a survivor’s conceptualization of the experience [25], resulting in underreporting. Further, many typologies of violence are considered concentrically inclusive, with experiences such as “rape” (widely defined as penetrative sex without freely given consent) encompassed by “sexual assault” (any form of sexual violence involving physical contact), which is then encompassed by “sexual violence” inclusive of contact and non-contact typologies. It has been suggested that using multiple measures, with questions asked in several ways, is needed to understand true prevalence [25]. Similarly, it has been suggested that the survivor’s conceptualization, self-definition, or understanding of SV is more influential and predictive of later impacts than behaviorally specific classifications [27–30]. With this in mind, three SV variables were employed in this analysis. First, *past-month forced/threatened sex* was assessed for study inclusion criteria, using two behaviorally-specific, time-bound questions: (1) “In the past month, has a man used force (like hitting, holding down, or using a weapon) to make you have vaginal sex?” and (2) “In the past month, has a man used threats to make you have vaginal sex when you did not want to?” Respondents indicating “yes” were offered enrollment in the study as “case” participants who had experienced past-month forced/threatened sex. *Previous forced/threatened sex* was measured using eight behaviorally specific items, which followed the format “Has (a (male/female) sex partner/any other (male/female)) ever used (force/threats) to make you have sex when you did not want to?”. “Case” participants were asked the same question, with the caveat, “Other than this past month…” Participants indicating yes to any of the questions were classified as having experienced *previous forced/threatened sex*; while questions were asked for both male and female perpetrators, all respondents indicated perpetration by only males or by both males and females. Finally, *lifetime sexual assault* was captured using one item from the Lifetime Events Checklist [31], in which participants were asked if “Sexual Assault (rape, attempted rape, made to perform any type of sexual act through force or threat of harm)” had happened to them. While examples of experiences falling within the category of “sexual assault” were provided, participants were encouraged to self-define sexual assault based on their experiences, as has been argued for to empower survivors of violence and decrease invalidation of experiences [25]. Participants indicating yes to this question were classified as having experienced sexual assault. “Case” participants were not asked this question due to efforts to limit potentially re-traumatizing content; as survivors of completed rape, which was one of the provided examples of sexual assault, these women were automatically classified as having experienced sexual assault.

Medical mistrust (MM) was captured using the Group-Based Medical Mistrust Scale [32], the most commonly used assessment of MM [12, 33], which has demonstrated adequate convergent validity [32, 34]. The scale consists of 11 prompts with which participants are asked to rate their agreement on a five-point Likert scale ranging from “Strongly Disagree” to “Strongly Agree.” Four items were reverse coded, then all items were summed to create a continuous scale with scores ranging from 12 to 60. Additionally, the scale was analyzed as three factor-derived subscales: *suspicion* (6 items), *discrimination* (3 items, also known as “group disparities in health care”), and *lack of support* (3 items) [32]. For subscales, appropriate items were reverse coded and summed to create a continuous subscale, with scores ranging 6 to 30 (suspicion subscale) and 3 to 15 (discrimination and lack of support subscales).

### Analysis

Univariate analyses (percentages, means) were used to describe the sample. For comparative analyses, the full sample was compared using independent t-tests to assess between-group differences in MM scores by the independent variables of interest: dichotomized race (Black vs. non-Black, White vs. non-White, Asian or Pacific Islander (API) vs. non-API), dichotomized ethnicity (Hispanic/Latina vs. non-Hispanic/Latina), and dichotomized sexual violence history (past-month forced sex vs. none, previous forced sex vs. none, lifetime exposure to self-defined sexual assault vs. none). Both the full MM scale and MM subscales were analyzed as outcome variables. Additionally, within-group differences in the relationship between dichotomized sexual violence history and MM was assessed among participants identifying with each racial or ethnic group. All means were reported with standard deviations; two-sided p-values were reported for comparative analyses. Variances were assumed equal except when indicated unequal by Levene’s test for equality of variances; p-values were reported in line with these assumptions. Cohen’s *d* was reported as an indicator of independent samples effect sizes, due to the difficulty of comparing full and subscale analyses. Cohen’s *d* was considered to indicate effect size of small (|*d*|*<0.20)*, medium (|*d*|*<0.50)*, or large (|*d*|*<0.80)* based on the existing literature [35, 36].

## Results

Sixty-eight individuals enrolled in *The THRIVE Study*, including 25 “case” participants who had experienced past-month forced or threatened vaginal sex, and 43 “control” participants who had experienced recent consensual vaginal sex (Table 1). Participants had a median age of 21.5 (IQR:9), with participants identifying as Black/African American (23.5%), White (36.8%), Asian or Pacific Islander (22.1%), and Hispanic/Latina (42.6%); 29.4% of these participants identified with more than one racial and ethnic category. Most participants had completed high school, received a GED, or less (55.9%) and were employed part-time (45.6%). Three-quarters of participants had experienced what they identified as sexual assault in their lifetime, while 36.8% had experienced previous forced or threatened sex; of these, two participants indicated experiences with both men and women as perpetrators, while the remainder indicated men only as perpetrators. The mean MM overall score was 28.22 (SD: 9.54, range: 12–51), while mean scores for MM sub-scales were 12.54 (SD:5.01, range: 6–23) for suspicion, 8.50 (SD:3.37, range: 3–15) for discrimination, and 7.18 (SD:2.76, range: 3–14) for lack of support; for the total scale and each subscale, higher scores indicate higher levels of MM.

**Table 1:** Study participant Characteristics, San Diego, CA 2018 to 2020 (*N* = 68)

Variable *N* (%), µ (SD), or Median (IQR)	
Age (years)	21.5 (9)
**Race** (Not Mutually Exclusive)	
Black/African American	16 (23.5)
White	25 (36.8)
Asian or Pacific Islander	15 (22.1)
Multiracial	20 (29.4)
**Ethnicity**	
Hispanic/Latina	29 (42.6)
**Educational Attainment**	
High School Diploma, GED, or Less	38 (55.9)
Some Trade, Vocational School, or College or more	30 (44.1)
**Employment Status**	
Unemployed	22 (32.4)
Employed Part-Time	31 (45.6)
Employed Full-Time	15 (22.1)
**Violence History** (Not Mutually Exclusive)	
Lifetime Sexual Assault	51 (75.0)
Previous Forced/Threatened Sex	31 (45.6)
Past-Month Forced/Threatened Sex	25 (36.8)
**Medical Mistrust**	
Total	28.22 (9.54)
Suspicion Subscale	12.54 (5.01)
Discrimination Subscale	8.50 (3.37)
Lack of Support Subscale	7.18 (2.76)

Note: Lifetime sexual assault can include recent or previous forced/threatened sex; “previous” forced/threatened sex does not include past-month forced/threated sex

Total MM score was significantly higher among women identifying as Black/African American versus all other women (*µ* = 33.94, SD: 9.39 vs. *µ* = 26.35, SD: 9.00, *p* = 0.005; Table 2) with a strong effect size (*d*=−0.834), as well as significantly higher scores on the *suspicion* (*µ* = 15.69, SD: 5.30 vs. *µ =* 11.58, SD: 4.55, *p* = 0.003) and *lack of support* (*µ* = 9.25, SD: 2.54 vs. *µ =* 6.51, SD: 2.54, p = < 0.001) subscales (Table 3). White individuals had a significantly lower *lack of support* subscale score compared to all other participants (*µ* = 6.24, SD: 2.35 vs. *µ = 7.72*, SD: 2.86, *p* = 0.032).

Women who had experienced previous forced/threatened sex had significantly higher total MM than those who had not (*µ* = 31.00, SD: 9.06 vs. *µ = 25.89*, SD: 9.41, *p* = 0.027) as did women who had experienced past-month forced/threatened sex (*µ* = 32.28, SD: 8.97 vs. *µ =* 25.86, SD: 9.15, *p* = 0.006), both with medium effect sizes (*d=*−0.552 and *d* = 0.707, respectively; Table 2). While women who had experienced previous forced/threatened sex had significantly higher scores on the *discrimination* subscale (*µ* = 9.48, SD: 3.38 vs. *µ =* 7.68, SD: 3.20, *p* = 0.027), women who had experienced past-month forced/threatened sex had significantly higher scores on the *suspicion* (*µ* = 14.96, SD: 4.57 vs. *µ =* 11.14, SD: 4.76, *p* = 0.002) and *lack of support* (*µ* = 8.08, SD: 2.80 vs. *µ =* 6.65, SD: 2.64, *p* = 0.039) subscales (Table 3).

**Table 2:** Medical mistrust by demographic group and by violence history, San Diego, CA 2018 to 2020 (*N* = 68)

Race		Medical Mistrust Score, µ (SD)	*P*-value	Cohen’s d
Hispanic/Latina	Yes	27.86 (9.53)	0.791	0.065
	No	28.49 (9.66)		
Asian or Pacific Islander	Yes	25.73 (9.82)	0.269	0.327
	No	28.87 (9.512)		
Black/African American	Yes	33.94 (9.39)	0.005	−0.834
	No	26.35 (9.00)		
White	Yes	26.08 (8.17)	0.130	0.358
	No	29.47 (10.13)		
Multiracial	Yes	28.95 (9.57)	0.665	−0.116
	No	27.83 (9.69)		
*SV*		*Medical Mistrust Score*,* µ (SD)*	*P-value*	*Cohen’s d*
Lifetime Sexual Assault	Yes	29.14 (9.63)	0.172	−0.387
	No	25.47 (8.97)		
Previous Forced/Threatened Sex	Yes	31.00 (9.06)	0.027	−0.552
	No	25.89 (9.41)		
Past Month Forced/Threatened Sex	Yes	32.28 (8.97)	0.006	0.707
	No	25.86 (9.15)		

**Table 3:** Medical mistrust subscale score by demographic group and by sexual violence history, San Diego, CA 2018 to 2020 (*N* = 68)

Race		Suspicion, µ (SD)	*p*	Discrimination, µ (SD)	*p*	Support, µ (SD)	*p*
Hispanic/Latina	Yes	12.14 (4.60)	0.569	8.55 (3.40)	0.976	7.17 (2.58)	0.983
	No	12.85 (5.34)		8.53 (3.43)		7.16 (2.96)	
Asian or Pacific Islander	Yes	11.00 (5.00)	0.179	7.67 (3.27)	0.262	7.07 (2.79)	0.879
	No	12.98 (4.98)		8.79 (3.41)		7.19 (2.81)	
Black/African American	Yes	15.69 (5.30)	0.003	9.0 (3.31)	0.536	9.25 (2.54)	< 0.001
	No	11.58 (4.55)		8.34 (3.44)		6.51 (2.54)	
White	Yes	11.36 (4.28)	0.139	8.54 (3.71)	0.971	6.24 (2.35)	0.032
	No	13.25 (4.50)		8.51 (3.22)		7.72 (2.86)	
Multiracial	Yes	13.00 (5.25)	0.632	8.35 (3.59)	0.770	7.60 (2.56)	0.407
	No	12.35 (4.95)		8.62 (3.34)		6.98 (2.88)	
*Violence History*		*Suspicion*,* µ (SD)*	*p*	*Discrimination*,* µ (SD)*	*p*	*Support*,* µ (SD)*	*p*
Lifetime Sexual Assault	Yes	13.12 (4.90)	0.103	8.65 (3.42)	0.538	7.37 (2.88)	0.315
	No	10.82 (5.10)		8.06 (3.31)		6.59 (2.35)	
Previous Forced/Threatened Sex	Yes	13.77 (4.61)	0.064	9.48 (3.38)	0.027	7.74 (2.90)	0.123
	No	11.51 (5.17)		7.68 (3.20)		6.70 (2.59)	
Past-Month Forced/Threatened Sex	Yes	14.96 (4.57)	0.002	9.24 (3.69)	0.170	8.08 (2.80)	0.039
	No	11.14 (4.76)		8.07 (3.15)		6.65 (2.64)	

Among women who identified as Hispanic/Latina, women who had endorsed experiencing each type of SV had significantly higher MM scores than those who did not, including for lifetime sexual assault (*µ* = 30.19, SD: 9.58 vs. *µ =* 21.75, SD: 6.48, *p* = 0.030), previous forced/threatened sex (*µ* = 35.25, SD: 7.85 vs. *µ =* 22.65, SD: 6.83, *p <* 0.001), and past-month forced/threatened sex (*µ* = 34.00, SD: 8.76 vs. *µ =* 22.88, SD: 6.99, *p* < 0.001) with large effect sizes for each (*d*=−0.951, *d*=−1.735, and *d* = 1.422, respectively; Table 4). Multiracial women who endorsed experiencing previous forced/threatened sex had significantly higher MM compared to Multiracial women who did not (*µ* = 33.30, SD: 9.60 vs. *µ =* 24.60, SD: 7.69, *p* = 0.038) with a small effect size (*d*=−0.100), while Multiracial survivors of past-month forced/threatened sex had significantly higher MM compared to their counterparts (*µ* = 33.54, SD: 8.49 vs. *µ =* 20.43, SD: 3.95, *p* = 0.001, *d* = 1.796). Similarly, White women who experienced past-month forced/threatened sex had significantly higher MM compared to White women with no such experiences (*µ* = 30.75, SD: 8.05 vs. *µ =* 21.77, SD: 5.67, *p* = 0.004) *d* = 1.298. There were no significant differences by SV experience among Black/African American women or Asian or Pacific Islander women.

**Table 4:** Medical mistrust (µ (SD)) by sexual violence history within racial/ethnic Group, San Diego, CA 2018 to 2020 (*N* = 68)

*Lifetime Sexual Assault*				
*Race/Ethnicity*	*Yes*	*No*	*P-value*	*Cohen’s d*
Hispanic/Latina	30.19 (9.58)	21.75 (6.48)	0.030	−0.951
Asian or Pacific Islander	25.75 (11.01)	25.67 (3.06)*	0.990	−0.008
Black/African American	35.45 (7.58)	30.60 (12.95)	0.355	−0.515
White	26.86 (8.37)	22.00 (6.38)*	0.285	−0.597
Multiracial	30.59 (9.46)	19.67 (1.53)*	0.067	−1.222
*Previous Forced/Threatened Sex*				
*Race/Ethnicity*	*Yes*	*No*	*P-value*	*Cohen’s d*
Hispanic/Latina	35.25 (7.85)	22.65 (6.83)	< 0.001	−1.735
Asian or Pacific Islander	26.60 (10.64)	25.80 (9.99)	0.972	0.020
Black/African American	34.00 (10.34)	33.88 (9.06)	0.490	−0.013
White	28.21 (7.59)	23.36 (8.43)	0.144	−0.609
Multiracial	33.30 (9.60)	24.60 (7.69)	0.038	−0.100
*Past-Month Forced/Threatened Sex*				
*Race/Ethnicity*	*Yes*	*No*	*P-value*	*Cohen’s d*
Hispanic/Latina	34.00 (8.76)	22.88 (6.99)	< 0.001	1.422
Asian or Pacific Islander	29.20 (14.11)	24.00 (7.18)	0.353	0.528
Black/African American	33.36 (10.97)	35.2 (5.21)	0.657	0.190
White	30.75 (8.05)	21.77 (5.67)	0.004	1.298
Multiracial	33.54 (8.49)	20.43 (3.95)	0.001	1.796

* Sub-sample size < 5

Among Hispanic/Latina women, experiences of previous and past-month forced/threatened sex were significantly associated with higher MM across all domains (*p* < 0.001–0.019), while experience of lifetime sexual assault was only associated with higher scores in the MM *suspicion* domain (*µ* = 13.52, SD: 4.52 vs. *µ =* 8.50, SD: 2.33, *p* < 0.001; Table 5.). Among White women, past-month forced/threatened was associated with higher MM in the *suspicion* (*µ* = 14.00, SD: 3.95 vs. *µ =* 8.92, SD: 2.99, *p* = 0.001) and *lack of support* domains (*µ* = 7.33, SD: 2.46 vs. *µ =* 5.32, SD: 1.79, *p* = 0.022). Among multiracial women, MM scores in the *suspicion* domain were significantly associated with experience of past-month forced/threatened sex (*µ* = 15.77, SD: 4.37 vs. *µ =* 7.86, SD: 2.27, *p* < 0.001), while *discrimination* domain scores were significantly associated with all types of SV (*p* = 0.008–0.028).

**Table 5:** Medical mistrust subscale score within Racial/Ethnic group by sexual violence experience

*Hispanic/Latina*							
Violence History		Suspicion, µ (SD)	*p*	Discrimination, µ (SD)	*p*	Support, µ (SD)	*p*
Lifetime Sexual Assault	Yes	13.52 (4.52)	< 0.001	9.04 (3.57)	0.209	7.62 (2.60)	0.133
	No	8.50 (2.33)		7.25 (2.66)		6.00 (2.27)	
Previous Forced/Threatened Sex	Yes	15.50 (4.30)	< 0.001	11.00 (3.10)	< 0.002	8.75 (2.38)	0.004
	No	9.76 (3.15)		6.82 (2.43)		6.05 (2.14)	
Past Month Forced/Threatened Sex	Yes	15.23 (4.21)	< 0.001	10.15 (3.78)	0.019	8.62 (2.40)	0.004
	No	9.63 (3.22)		7.25 (2.46)		6.00 (2.13)	
*Asian or Pacific Islander*							
*Violence History*		*Suspicion*,* µ (SD)*	*p*	*Discrimination*,* µ (SD)*	*p*	*Support*,* µ (SD)*	*p*
Lifetime Sexual Assault	Yes	11.08 (5.43)	0.903	7.50 (3.23)	0.708	7.17 (3.01)	0.793
	No	10.67 (3.51)*		8.33 (4.04)*		6.67 (2.08)*	
Previous Forced/Threatened Sex	Yes	11.80 (5.67)	0.678	6.60 (2.88)	0.391	7.20 (2.86)	0.901
	No	10.60 (4.90)		8.20 (3.46)		7.00 (2.91)	
Past Month Forced/Threatened Sex	Yes	13.2 (5.76)	0.242	8.40 (4.51)	0.558	7.60 (3.97)	0.619
	No	9.90 (4.48)		7.30 (2.67)		6.80 (2.20)	
*Black/African American*							
*Violence History*		*Suspicion*,* µ (SD)*	*p*	*Discrimination*,* µ (SD)*	*p*	*Support*,* µ (SD)*	*p*
Lifetime Sexual Assault	Yes	16.09 (4.30)	0.667	9.45 (3.50)	0.434	9.91 (2.26)	0.128
	No	14.80 (7.60)		8.00 (2.92)		7.80 (2.77)	
Previous Forced/Threatened Sex	Yes	14.63 (4.87)	0.442	9.38 (3.74)	0.666	10.00 (2.44)	0.251
	No	16.75 (5.82)		8.63 (3.02)		8.50 (2.56)	
Past Month Forced/Threatened Sex	Yes	17.40 (3.51)	0.402	8.20 (3.27)	0.533	9.60 (2.88)	0.724
	No	14.91 (5.92)		9.36 (3.41)		9.09 (2.51)	
*White*							
*Violence History*		*Suspicion*,* µ (SD)*	*p*	*Discrimination*,* µ (SD)*	*p*	*Support*,* µ (SD)*	*p*
Lifetime Sexual Assault	Yes	11.90 (4.43)	0.149	8.57 (3.63)	0.784	6.38 (2.54)	0.173
	No	8.50 (1.73)*		8.00 (4.69)*		5.50 (0.58)*	
Previous Forced/Threatened Sex	Yes	12.29 (4.12)	0.230	9.57 (3.63)	0.098	6.36 (2.59)	0.785
	No	10.18 (4.38)		7.09 (3.48)		6.09 (2.12)	
Past Month Forced/Threatened Sex	Yes	14.00 (3.95)	0.001	9.42 (3.94)	0.233	7.33 (2.46)	0.022
	No	8.92 (2.99)		7.62 (3.40)		5.23 (1.79)	
*Multiracial*							
*Violence History*		*Suspicion*,* µ (SD)*	*p*	*Discrimination*,* µ (SD)*	*p*	*Support*,* µ (SD)*	*p*
Lifetime Sexual Assault	Yes	13.88 (5.18)	0.072	8.76 (3.75)	0.008	7.94 (2.63)	0.162
	No	8.00 (1.73)*		6.00 (0.00)*		5.67 (0.58)*	
Previous Forced/Threatened Sex	Yes	14.50 (4.77)	0.210	10.10 (3.98)	0.028	8.70 (2.63)	0.052
	No	11.50 (5.52)		6.60 (2.12)		6.50 (2.07)	
Past Month Forced/Threatened Sex	Yes	15.77 (4.37)	< 0.001	9.38 (4.05)	0.027	8.38 (2.63)	0.060
	No	7.86 (2.27)		6.43 (1.13)		6.14 (1.77)	

* Sub-sample size < 5

## Discussion

We sought to understand differences in MM by race and ethnicity and SV survivorship status. Overall, women who had experienced previous or past-month forced or threatened sex had significantly higher MM than those without each experience, with women who experienced past-month forced/threatened sex having a mean MM score 1.28 points higher than women with previous experiences. For women with previous experience of forced/threatened sex, significant differences were present in the *discrimination* domain, while women with past-month experience had significantly higher scores in the *suspicion* and *lack of support* domains. This may suggest an acute MM reaction centering on medical providers not believing women following circumstances of rape [37, 38] and thereby not providing adequate service linkage or help; correspondingly, these findings may also indicate insufficient training of health care providers on responding to sexual assault, sensitivity following assault, survivor-validating care, and needs of survivors follow sexual assault [39]. For non-recent survivors of SV, retrospective examination of experiences related to SV, and the responsiveness or lack thereof of providers to SV (including screening, validation, provision of services, linkage, etc.) may shift this mistrust to being discrimination-centered; in particular, efforts to understand negative medical experiences may point to discrimination based on race, ethnicity, gender, survivor status, sexual orientation, or other factors.

Importantly, forced/threatened sex is associated with higher MM regardless of when it occurred. These lasting impacts must be considered; particularly in that a patient may be a survivor of forced sex- with significant MM associated with that violence- regardless of if they present for care related to SV. This is of particular concern in primary care settings, where survivors of violence may be less likely to seek post-assault care or disclose violence histories [10], and where physical exams may unknowingly mimic circumstances of assault (physical touch, lack of choice, etc.).

In addition to significant associations with forced sex, in this sample, sexual assault as a larger category defined as “rape, attempted rape, made to perform any type of sexual act through force or threat of harm” was associated with increased MM for some women. This suggests that the qualities of SV may be pertinent for some demographic groups as they relate to MM or MM domains, but for other women, any experience of sexual assault may be sufficiently influential to impact care interactions. Application of patient-centered and trauma-informed practices should therefore not be limited to those who disclose forced/threatened sex (rape), but all survivors of sexual assault. Further, there are myriad types of SV beyond physical sexual assault, including coercion, non-contact SV, and technological-facilitated SV, among others. While not sufficiently captured for analysis in this study, the potential impact of these experiences on MM and health care seeking should not be ignored.

To both improve post-violence care seeking for future women who experience SV, as well as improve provider-patient trust, it is essential that medical providers implement patient-centered and trauma-informed practices in regular care. This is particularly important for primary care providers, who may serve as gatekeepers to specialized care and insurance coverage of care [40]. Such practices may include validation of experiences, survivor-led provision of service linkages, and employment of shared decision-making. Of National Domestic Violence Hotline callers surveyed in 2022, the majority said that would be more comfortable seeking medical care if they knew a provider would believe them (68.8%), that the provider(s) practiced trauma-informed care (66.7%), that patients could trust the staff (62.0%; *n* = 173), and that patients were guaranteed confidentiality (59.5%; *n* = 166) [5]. However, a recent scoping review identified wide variety in training programs; while health care professionals reported improvements in knowledge and confidence to discuss SV, changes in clinical care reflective of these were seldom measured [41], though some studies have seen increased distal outcomes, such as disclosures and referral to services [42]. The authors conclude that a mix of participatory methods (ex., roleplay) and passive didactic methods (ex., lectures) were most effective for health care professionals to improve knowledge and confidence, as well as reiteration to reinforce knowledge and skills [41]. Of stylized interventions for survivors of violence in primary care, many focus on “treatment” and support of recovery [43, 44] as opposed to increased acceptability of care and survivor-led engagement in support services. While violence-specific support may be vital for some survivors, others may be seeking empathetic and acceptable care that is agnostic to “treatment” but allows for engagement in care for other primary care concerns or routine wellness visits without re-traumatization.

For Hispanic/Latina women, total MM and MM subscores were significantly influenced by SV history, particularly previous or past-month experiences of forced/threatened sex; lifetime sexual assault was associated only with higher MM within the *suspicion* domain. For multiracial women, previous and past-month experiences of SV were significantly associated with total MM, with sub-analyses indicating that these experiences were associated with increased MM in the *discrimination* domain, specifically, while past-month forced/threatened sex was also associated with higher MM within the *suspicion* domain. White women had overall lower levels of perceived *lack of support*, though White women who experienced past-month forced/threatened sex had higher MM that those who did not, with significantly higher scores in the *suspicion* and *lack of support* domains. For these women, taking into account MM exacerbated by past-month experiences of SV may be vital for building trust and promoting engagement in care. This suggests that repeated violence screening in primary care settings is essential to facilitate informed engagement of patients in medical services, rather than one-time or infrequent screening. Much of the available literature focused on screening for IPV rather than other forms of violence; yet, a recent study in four hospital-associated primary care clinics found that even IPV screening only occurred at 8.5% of 400 primary care annual visits, compared to screening for anxiety (37.3%) and depression (71.3%) [45]. A 2015 Cochrane meta-analysis identified that screening for IPV in various health care settings did increase identification survivors [46], while in primary care settings for veterans, implementation of IPV screening was associated with 2.7 times the odds of being screened for IPV compared to pre-implementation. In this implementation trial, odds of disclosure of violence were equal among women screened pre-implementation and during implementation, but women screened during implementation were more likely to use psychological services after screening [47], indicating potential service uptake benefits beyond just the immediate care setting.

Such screening is important for the identification of recent survivors of violence who may be most in need of acute support or care related to SV; however, the variability in time frame of self-report screeners, and particularly the one-year time frame often utilized by screeners [48], may not allow for identification of those with a non-recent history of SV. This underscores the previously discussed importance of trauma-informed care for all patients, rather than only responsive to positive screens. Alternatively, a survivor may decline to disclose experiences in an effort to avoid stylized response, referral, or intervention for violence; these individuals also deserve trauma-informed and acceptable primary care.

Black women had the highest mean MM score of women in this sample. This score was significantly higher than non-Black women in the sample, but levels of mistrust among Black women were not significantly influenced by SV history. Given evidence that MM is associated with lower health care engagement [15] and research in other contexts that suggests violence is a barrier to engagement [17–19], this may indicate that medical settings are not an adequate setting to reach Black women for SV services or other social services. However, some research suggests that Black women who experience violence are more likely than others to engage in care [8]; help-seeking, both short- and long-term after violence, must be further explored to improve service delivery models for survivors of all racial and ethnic groups.

### Limitations

Little research has explored SV, and specifically subtypes of SV, as they relate to MM and potential medical care engagement. Further analysis of these relationships within racial and ethnic groups allows for targeted efforts to improve patient-provider relationships or otherwise meet survivors of violence “where they are.” Because of the high prevalence of endorsement of sexual assault, some racial and ethnic groups had cells sized below 5 for sub-group analyses; as a result, significant effects may have been missed due to a lack of power. It is possible that significant associations identified are spurious in nature, but the consistency of findings within and across sub-group analyses strengthens confidence that they reflect real differences between groups. Data on experiences of sexual violence was self-reported by participants, leading to possible recall or social desirability biases; however, women were able to be enrolled regardless of history of sexual violence due to the study design, limiting any perceive incentive to reporting events that did not occur; further, under-reporting of sexual violence is widely understood to be more likely that reporting of events that did not occur, lending added likehood of veracity of reported events. Not all of the measures used to asses violence were behaviorally specifically, which could lead to misclassification of experiences; however, some research suggests that self-definition, conceptualization, or understanding of SV is more influential than behaviorally-specific classifications [27–30], and therefore we believe the multiple measure methodology used in this analysis is a strength.

## Conclusions

Our study findings highlight the critical need for provider level interventions that promote trauma-informed care training, especially in the context of MM and the domains of discrimination and suspicion. Our study also adds to the extant literature on MM among survivors of SV, offering recommendations for future research with longer follow-up time periods and larger samples. Nonetheless, our current findings can facilitate the development and testing of programs in a healthcare setting.

## Supplementary Information

Supplementary Material 1.

## Data Availability

The datasets analyzed during the current study are not publicly available due the sensitivity of the research, but are available from the corresponding author on reasonable request.

## Abbreviations

AI/AN: American Indian/Alaska Native
CA: California
IPV: Intimate Partner Violence
IQR: Interquartile Range
MM: Medical Mistrust
SD: Standard Deviation
STIs: Sexually Transmitted Infections
SV: Sexual Violence
U.S.: United States
