# Supplementary material for: Medical mistrust among diverse survivors of sexual violence: implications for primary health care delivery and engagement
Source: BMC Prim Care. 2025 Dec 15;26:397. doi: 10.1186/s12875-025-03105-4 (PMC12706906; doi:10.1186/s12875-025-03105-4)
Supplement: Supplementary file 1 — Supplementary Material 1. [file 12875_2025_3105_MOESM1_ESM.pdf]

Baseline Survey

Please complete the survey below.

Thank you!

What study group is this participant a member of?	◯ Case (Sexual Assault)<br>◯ Control (Sexually Active)
Was this survey conducted in-person or remotely?	◯ In-Person<br>◯ Remotely (Phone Call, Zoom)

Section 1: Personal History

SAY: Thank you for agreeing to participate in this study. This survey will ask you questions about your medical history, reproductive health and sexual history, and day-to-day experiences. All of your responses will be kept confidential, and you do not have to answer any questions that you are uncomfortable with.

These first questions are going to ask a little bit about you and your current situations. Please provide the answers that are most appropriate for you.

Demographics

What is your date of birth?

____

Do you consider yourself to be Hispanic or Latina?

(Meaning a person of Cuban, Mexican, Puerto Rican, South or Central American, or other Spanish origin regardless of race)

- ◯ Yes
- ◯ No

How do you describe your racial background?

- [ ] American Indian or Alaska Native
- [ ] Asian
- [ ] Black / African American
- [ ] Native Hawaiian or other Pacific Islander
- [ ] White
- [ ] Other

NOTES:

What are the definitions of the federal race categories?

American Indian or Alaska Native: A person having origins in any of the original peoples of North and South America (including Central America), and who maintains a tribal affiliation or community attachment.

Asian: A person having origins in any of the original peoples of the Far East, Southeast Asia, or the Indian subcontinent including, for example, Cambodia, China, India, Japan, Korea, Malaysia, Pakistan, the Philippine Islands, Thailand, and Vietnam.

Black or African American: A person having origins in any of the Black racial groups of Africa.
Native Hawaiian or Other Pacific Islander: A person having origins in any of the original peoples of Hawaii, Guam, Samoa, or other Pacific Islands.

White: A person having origins in any of the original peoples of Europe, the Middle East, or North Africa.

There doesn't seem to be a race category to select when respondents select their ethnicity as Hispanic/Latino. What's the correct race selection for someone who is Hispanic or Latino?

The federal government considers "Hispanic/Latino" to be an ethnicity, not a race. Consequently, "Hispanic/Latino" is not a selection in the race part of the question. We support self-identification, so the correct answer is whatever the person indicates in both parts of the question. Someone who is Hispanic or Latino could see themselves as belonging to any one or more than one race. We understand that some respondents might not identify themselves as belonging to any of the race groups if they identify their ethnicity as Hispanic/Latino. If a respondent does not see a race group that applies after selecting Hispanic/Latino ethnicity, and after the definitions or other help has been provided, the respondent might leave the race part blank.

Other, please specify: ______________________________

In what country where you born? ______________________________

What is the highest level of education that you have completed?	◯ 8th grade or less<br>◯ 9th grade or more but did not complete high school or receive a GED<br>◯ Graduated from high school or received a GED<br>◯ Some trade school, vocational school, or some college<br>◯ Completed trade school or vocational school<br>◯ Completed an associate's degree<br>◯ Completed a bachelor's degree<br>◯ Completed a graduate degree (e.g. master, professional, and/or doctoral degree)<br>◯ Other (please specify):
Are you currently a student?	◯ Yes<br>◯ No
What level of student are you currently?	◯ Middle School<br>◯ High School<br>◯ Trade/Vocational School<br>◯ Community College (e.g. 2 year program)<br>◯ College/University (e.g. 4 year program)<br>◯ Graduate School (e.g. master, professional, and/or doctoral degree)
Other, please specify:	__________________________________
What is currently your sexual orientation?	◯ Straight or Heterosexual<br>◯ Gay or Lesbian<br>◯ Bisexual<br>◯ Other<br>◯ Unsure<br>◯ Prefer not to answer
Other, please specify:	__________________________________
What is your marital status?	◯ Single<br>◯ Partnered, but not married<br>◯ Married<br>◯ Separated<br>◯ Divorced<br>◯ Widowed<br>◯ Other
Other, please specify:	__________________________________
Are you currently employed or self-employed?	◯ Yes<br>◯ No
Do you work full-time or part-time?	◯ Full-Time<br>◯ Part-Time<br>◯ Self-Employed
Do you have children and/or dependents under the age of 18?	◯ Yes<br>◯ No

How many children and/or dependents do you have under the age of 18?

____

Which of the following best describes your current living situation? By current living situation I mean where you have been staying during the past seven days. (Mark all that apply.)

- ☐ Your own place, a room, apartment, or a house that is your home
- ☐ Temporarily doubled up with others, in someone else's house, apartment, or room
- ☐ A temporary or transitional housing program
- ☐ SRO, that is a single room occupancy facility, or a welfare hotel or motel
- ☐ In a shelter for homeless people
- ☐ In jail, prison, or a halfway house
- ☐ In drug treatment, a detox unit, or drug program housing
- ☐ In a hospital, nursing home, or hospice
- ☐ In an abandoned building, a public place like a bus station, a store or another place
- ☐ On the street or anywhere outside such as a park, under a bridge or in a campground
- ☐ Someplace else
- ☐ Do not want to answer
- ☐ Don't know/ Don't remember

Other, please specify:

____

Where did you sleep last night?

- ☐ Your own place, a room, apartment, or a house that is your home
- ☐ Temporarily doubled up with others, in someone else's house, apartment, or room
- ☐ A temporary or transitional housing program
- ☐ SRO, that is a single room occupancy facility, or a welfare hotel or motel
- ☐ In a shelter for homeless people
- ☐ In jail, prison, or a halfway house
- ☐ In drug treatment, a detox unit, or drug program housing
- ☐ In a hospital, nursing home, or hospice
- ☐ In an abandoned building, a public place like a bus station, a store or another place
- ☐ On the street or anywhere outside such as a park, under a bridge or in a campground
- ☐ Someplace else
- ☐ Do not want to answer
- ☐ Don't know/ Don't remember

Other, please specify:

____

Do you currently receive any of the following?

- ☐ Public housing, housing assistance, housing vouchers
- ☐ Medicaid
- ☐ WIC
- ☐ Food stamps
- ☐ Disability, SSI, SSDI
- ☐ Other public assistance
- ☐ None of these
- ☐ Do not want to answer
- ☐ Don't know/ Don't remember

Other, please specify: __________________________________

Which category best describes your current annual (yearly) income from all sources?

- ◯ Less than $10,000
- ◯ $10,000 - $19,999
- ◯ $20,000 - $29,999
- ◯ $30,000 - $39,999
- ◯ $40,000 - $49,999
- ◯ $50,000 or more
- ◯ Do not want to answer
- ◯ Don't know/ Don't remember

Section 2: Mental Health History

SAY: For the next few questions, I would like you to think about how you felt prior to one week ago.

Prior to one week ago, had you ever had any experience that was so frightening, horrible, or upsetting that you:<br><br>Have had nightmares about it or thought about it when you did not want to?	◯ Yes<br>◯ No
Tried hard not to think about it or went out of your way to avoid situations that reminded you of it?	◯ Yes<br>◯ No
Were constantly on guard, watchful, or easily startled?	◯ Yes<br>◯ No
Felt numb or detached from others, activities, or your surroundings?	◯ Yes<br>◯ No

SAY: In your life, have you ever had any experience that was so frightening, horrible, or upsetting that, in the past month, you:

Have had nightmares about it or thought about it when you did not want to?	◯ Yes<br>◯ No
Tried hard not to think about it or went out of your way to avoid situations that reminded you of it?	◯ Yes<br>◯ No
Were constantly on guard, watchful, or easily startled?	◯ Yes<br>◯ No
Felt numb or detached from others, activities, or your surroundings?	◯ Yes<br>◯ No

The following questions address your feelings prior to one week ago. Please indicate how frequently you felt in the following ways. (RESPONSE CARD PAGE 1)

	Rarely or none of the time (less than 1 day per week)	Some or a little of the time (1-2 days per week)	Occasionally or a moderate amount of time (3-4 days per week)	All of the time (5-7 days per week)
I was bothered by things that usually don't bother me.	○	○	○	○
I had trouble keeping my mind on what I was doing.	○	○	○	○
I felt depressed.	○	○	○	○
I felt that everything I did was an effort.	○	○	○	○
I felt hopeful about the future	○	○	○	○
I felt fearful.	○	○	○	○
My sleep was restless.	○	○	○	○
I was happy.	○	○	○	○
I felt lonely.	○	○	○	○
I could not "get going."	○	○	○	○

The following questions address your feelings in the past week. Please indicate how frequently you felt in the following ways. (RESPONSE CARD PAGE 1)

	Rarely or none of the time (less than 1 day per week)	Some or a little of the time (1-2 days per week)	Occasionally or a moderate amount of time (3-4 days per week)	All of the time (5-7 days per week)
I was bothered by things that usually don't bother me.	○	○	○	○
I had trouble keeping my mind on what I was doing.	○	○	○	○
I felt depressed.	○	○	○	○
I felt that everything I did was an effort.	○	○	○	○
I felt hopeful about the future.	○	○	○	○
I felt fearful.	○	○	○	○
My sleep was restless.	○	○	○	○
I was happy.	○	○	○	○
I felt lonely.	○	○	○	○
I could not "get going."	○	○	○	○

Thinking about prior to one week ago, how often did you feel the following ways? (RESPONSE CARD PAGE 2)

	Never	Almost Never	Sometimes	Fairly Often	Very Often
How often have you been upset because of something that happened unexpectedly?	○	○	○	○	○
How often have you felt that you were unable to control the important things in your life?	○	○	○	○	○
How often have you felt nervous and "stressed"?	○	○	○	○	○
How often have you felt confident about your ability to handle your personal problems?	○	○	○	○	○
How often have you felt that things were going your way?	○	○	○	○	○
How often have you found that you could not cope with all the things you had to do?	○	○	○	○	○
How often have you been able to control irritations in your life?	○	○	○	○	○
How often have you felt that you were on top of things?	○	○	○	○	○
How often have you been angered because of things that were outside of your control?	○	○	○	○	○
How often have you felt that difficulties were piling up so high that you could not overcome them?	○	○	○	○	○

The following questions address your feelings in the past week. Please indicate how frequently you felt the following ways.
(RESPONSE CARD PAGE 2)

	Never	Almost Never	Sometimes	Fairly Often	Very Often
How often have you been upset because of something that happened unexpectedly?	○	○	○	○	○
How often have you felt that you were unable to control the important things in your life?	○	○	○	○	○
How often have you felt nervous and "stressed"?	○	○	○	○	○
How often have you felt confident about your ability to handle your personal problems?	○	○	○	○	○
How often have you felt that things were going your way?	○	○	○	○	○
How often have you found that you could not cope with all the things you had to do?	○	○	○	○	○
How often have you been able to control irritations in your life?	○	○	○	○	○
How often have you felt that you were on top of things?	○	○	○	○	○
How often have you been angered because of things that were outside of your control?	○	○	○	○	○
How often have you felt that difficulties were piling up so high that you could not overcome them?	○	○	○	○	○

Thinking about prior to one week ago, please indicate how frequently you felt the following ways.
(RESPONSE CARD PAGE 3)

	Not true at all	Rarely true	Sometime true	Often true	True nearly all the time
I am able to adapt to change.	○	○	○	○	○
I can deal with whatever comes.	○	○	○	○	○
I try to see the humorous side of problems.	○	○	○	○	○
Coping with stress can strengthen me.	○	○	○	○	○
I tend to bounce back after illness or hardship.	○	○	○	○	○
I can achieve goals despite obstacles.	○	○	○	○	○
I can stay focused under pressure.	○	○	○	○	○
I am not easily discouraged by failure.	○	○	○	○	○
I think of myself as a strong person.	○	○	○	○	○
I can handle unpleasant feelings.	○	○	○	○	○

The following questions address your feelings in the past week. Please indicate how frequently you felt the following ways. (RESPONSE CARD PAGE 3)

	Not true at all	Rarely true	Sometimes true	Often true	True nearly all of the time
I am able to adapt to change.	○	○	○	○	○
I can deal with whatever comes.	○	○	○	○	○
I try to see the humorous side of problems.	○	○	○	○	○
Coping with stress can strengthen me.	○	○	○	○	○
I tend to bounce back after illness or hardship.	○	○	○	○	○
I can achieve goals despite obstacles.	○	○	○	○	○
I can stay focused under pressure.	○	○	○	○	○
I am not easily discouraged by failure.	○	○	○	○	○
I think of myself as a strong person.	○	○	○	○	○
I can handle unpleasant feelings.	○	○	○	○	○

Next I'm going to list a number of difficult or stressful things that sometimes happen to people. For each event indicated if (a) it happened to you personally; (b) you witnessed it happen to someone else; (c) you learned about it happening to a close family member or close friend; (d) you were exposed to it as part of your job (for example, paramedic, police, military, or other first responder); (e) you're not sure if it fits; or (f) it doesn't apply to you. Be sure to consider your entire life (growing up as well as adulthood) as you go through the list of events.

(RESPONSE CARD PAGE 4)

Natural Disaster (e.g. flood, hurricane, tornado, earthquake)

☐ Happened to me ☐ Witnessed it
☐ Learned about it ☐ Part of my job
☐ Not sure ☐ Doesn't Apply

Fire or explosion

☐ Happened to me ☐ Witnessed it
☐ Learned about it ☐ Part of my job
☐ Not sure ☐ Doesn't Apply

Transportation accident (e.g. car accident, boat accident, train wreck, plane crash)

☐ Happened to me ☐ Witnessed it
☐ Learned about it ☐ Part of my job
☐ Not sure ☐ Doesn't Apply

Serious accident at work, home, or during recreational activity

☐ Happened to me ☐ Witnessed it
☐ Learned about it ☐ Part of my job
☐ Not sure ☐ Doesn't Apply

Exposure to toxic substances (e.g. dangerous chemicals, radiation)

☐ Happened to me ☐ Witnessed it
☐ Learned about it ☐ Part of my job
☐ Not sure ☐ Doesn't Apply

Physical assault (e.g. being attacked, hit, slapped, kicked, beaten up)	☐ Happened to me ☐ Witnessed it ☐ Learned about it ☐ Part of my job ☐ Not sure ☐ Doesn't Apply
Assault with a weapon (e.g. being shot, stabbed, threatened with a knife, gun, bomb)	☐ Happened to me ☐ Witnessed it ☐ Learned about it ☐ Part of my job ☐ Not sure ☐ Doesn't Apply
Sexual Assault (rape, attempted rape, made to perform any type of sexual act through force or threat of harm)	☐ Happened to me ☐ Witnessed it ☐ Learned about it ☐ Part of my job ☐ Not sure ☐ Doesn't Apply
Unwanted or uncomfortable sexual experience other than a sexual assault.	☐ Happened to me ☐ Witnessed it ☐ Learned about it ☐ Part of my job ☐ Not sure ☐ Doesn't Apply
Combat or exposure to a war-zone (in the military or as a civilian)	☐ Happened to me ☐ Witnessed it ☐ Learned about it ☐ Part of my job ☐ Not sure ☐ Doesn't Apply
Captivity (e.g. being kidnapped, abducted, held hostage, prisoner of war)	☐ Happened to me ☐ Witnessed it ☐ Learned about it ☐ Part of my job ☐ Not sure ☐ Doesn't Apply
Life-threatening illness of injury	☐ Happened to me ☐ Witnessed it ☐ Learned about it ☐ Part of my job ☐ Not sure ☐ Doesn't Apply
Severe human suffering	☐ Happened to me ☐ Witnessed it ☐ Learned about it ☐ Part of my job ☐ Not sure ☐ Doesn't Apply
Sudden violent death (e.g. homicide, suicide)	☐ Happened to me ☐ Witnessed it ☐ Learned about it ☐ Part of my job ☐ Not sure ☐ Doesn't Apply
Sudden accidental death	☐ Happened to me ☐ Witnessed it ☐ Learned about it ☐ Part of my job ☐ Not sure ☐ Doesn't Apply
Serious injury, harm, or death you caused to someone else	☐ Happened to me ☐ Witnessed it ☐ Learned about it ☐ Part of my job ☐ Not sure ☐ Doesn't Apply
Any other very stressful event	☐ Happened to me ☐ Witnessed it ☐ Learned about it ☐ Part of my job ☐ Not sure ☐ Doesn't Apply

Here are 10 statements that other women have used to describe their lives with men/guys they have been involved with. This can include involvement sexually, emotionally, or otherwise, regardless of length of time or type of involvement (e.g. friend, acquaintance, casual dating, "talking," committed, etc). Please listen to each statement and indicate the answer that best describes how much you agree or disagree with each. Answer the questions thinking about your current or your most recent involvement with a man/guy.

(RESPONSE CARD PAGE 5)

	Strongly Agree	Somewhat Agree	Agree a Little	Disagree a Little	Somewhat Disagree	Strongly Disagree
He makes me feel unsafe even in my own home.	◯	◯	◯	◯	◯	◯
I feel ashamed of the things he does to me.	◯	◯	◯	◯	◯	◯
I try not to rock the boat because I am afraid of what he might do.	◯	◯	◯	◯	◯	◯
I feel like I am programmed to react a certain way to him.	◯	◯	◯	◯	◯	◯
I feel like he keeps me prisoner	◯	◯	◯	◯	◯	◯
He makes me feel like I have no control over my life, no power, no protection.	◯	◯	◯	◯	◯	◯
I hide the truth from others because I am afraid not to.	◯	◯	◯	◯	◯	◯
I feel owned and controlled by him.	◯	◯	◯	◯	◯	◯
He can scare me without laying a hand on me.	◯	◯	◯	◯	◯	◯
He has a look that goes straight through me and terrifies me.	◯	◯	◯	◯	◯	◯

Thinking about the previous set of questions, how would you describe the relationship you had with this person?

- ◯ Spouse
- ◯ Steady or main partner
- ◯ Casual dating partner
- ◯ Friend
- ◯ Acquaintance
- ◯ Co-worker
- ◯ Classmate
- ◯ Father
- ◯ Stepfather or my mother's boyfriend
- ◯ Other male family member
- ◯ Family Friend or Neighbor
- ◯ Stranger (someone you did not know)
- ◯ Other
- ◯ Don't want to answer

Other, please describe: ______________________________

Section 3: Sociocultural Factors

The next set of questions asks about your opinions on a number of statements. Please rate your agreement.
(RESPONSE CARD PAGE 6)

	Strongly Agree (1)	(2)	(3)	Undecided (4)	(5)	(6)	Strongly Disagree (7)
It is disrespectful for a man to swear in the presence of a lady.	○	○	○	○	○	○	○
Women should not expect men to offer them seats on buses	○	○	○	○	○	○	○
Homosexual relationships should be as socially acceptable as heterosexual relationships.	○	○	○	○	○	○	○
The initiation in courtship should usually come from the man.	○	○	○	○	○	○	○
It bothers me more to see a woman who is pushy than a man who is pushy.	○	○	○	○	○	○	○
When sitting down at the table, proper respect demands that the gentleman hold the lady's chair.	○	○	○	○	○	○	○
Women should have as much sexual freedom as men.	○	○	○	○	○	○	○
Women should appreciate the protection and support that men have traditionally given them.	○	○	○	○	○	○	○
Women with children should not work outside the home if they don't have to financially.	○	○	○	○	○	○	○
I see nothing wrong with a woman who doesn't like to wear skirts or dresses.	○	○	○	○	○	○	○
The husband should be regarded as the legal representative of the family group in all matters of law.	○	○	○	○	○	○	○
I like women who are outspoken.	○	○	○	○	○	○	○

Except perhaps in very special circumstances, a gentleman should never allow a lady to pay for the taxi, buy the tickets, or pay the check.	○	○	○	○	○	○	○
Some equality in marriage is good, but by and large the husband ought to have the main say-so in family matters.	○	○	○	○	○	○	○
Men should continue to show courtesies to women such as holding open the door or helping them on with their coats.	○	○	○	○	○	○	○
It is ridiculous for a woman to run a train and a man to sew clothes.	○	○	○	○	○	○	○
A woman should be as free as a man to propose marriage.	○	○	○	○	○	○	○
Women should be concerned with their duties of childbearing and house-tending, rather than with desires for professional and business careers.	○	○	○	○	○	○	○
Swearing and obscenity is more repulsive in the speech of a woman than a man.	○	○	○	○	○	○	○
There are some professions and types of businesses that are more suitable for men than women.	○	○	○	○	○	○	○

The questions address your current sexual relationships.

Please indicate how much you personally disagree or agree with each statement. (RESPONSE CARD PAGE 7)

	Strongly Disagree	Disagree	Agree	Strongly Agree
If I asked my partner to use a condom, he would get violent.	○	○	○	○
If I asked my partner to use a condom, he would be angry.	○	○	○	○
Most of the time, we do what my partner wants to do.	○	○	○	○
My partner won't let me wear certain things.	○	○	○	○
When my partner and I are together, I'm pretty quiet.	○	○	○	○
My partner has more say than I do about important decisions that affect us.	○	○	○	○
My partner tells me who I can spend time with.	○	○	○	○
If I asked my partner to use a condom, he would think I'm having sex with other people.	○	○	○	○
I feel trapped or stuck in our relationship.	○	○	○	○
My partner does what he wants, even if I do not want him to.	○	○	○	○
I am more committed to our relationship than my partner is.	○	○	○	○
When my partner and I disagree, he gets his way most of the time.	○	○	○	○
My partner gets more out of our relationship than I do.	○	○	○	○
My partner always wants to know where I am.	○	○	○	○
My partner might be having sex with someone else.	○	○	○	○

**Please indicate who makes decisions in your relationship.
(RESPONSE CARD PAGE 8)**

	Your Partner	Both of You	You
Who usually has more say about whose friends to go out with?	○	○	○
Who usually has more say about whether you have sex?	○	○	○
Who usually has more say about what you do together?	○	○	○
Who usually has more say about how often you see one another?	○	○	○
Who usually has more say about when you talk about serious things?	○	○	○
In general, who do you think has more power in your relationship?	○	○	○
Who usually has more say about whether you use condoms?	○	○	○
Who usually has more say about what types of sexual acts you do?	○	○	○

The next questions refer to your personal experiences and opinions of how you are treated. In your day-to-day life, how often do any of the following things happen to you? (RESPONSE CARD PAGE 9)

	Never	Less than once a year	A few times a year	A few times a month	At least once a week	Almost everyday
You are treated with less courtesy than other people are.	◯	◯	◯	◯	◯	◯
You are treated with less respect than other people are.	◯	◯	◯	◯	◯	◯
You receive poorer service than other people at restaurants or stores.	◯	◯	◯	◯	◯	◯
People act as if they think you are not smart.	◯	◯	◯	◯	◯	◯
People act as if they are afraid of you.	◯	◯	◯	◯	◯	◯
People act as if they think you are dishonest.	◯	◯	◯	◯	◯	◯
People act as if they're better than you are.	◯	◯	◯	◯	◯	◯
You are called names or insulted.	◯	◯	◯	◯	◯	◯
You are threatened or harassed.	◯	◯	◯	◯	◯	◯
You are followed around in stores	◯	◯	◯	◯	◯	◯

What do you think is the main reason for these experiences? (Check all that apply.)

- ☐ Your ancestry or national origins
- ☐ Your gender
- ☐ Your race
- ☐ Your age
- ☐ Your religion
- ☐ Your height
- ☐ Your weight
- ☐ Some other aspect of your physical appearance
- ☐ Your sexual orientation
- ☐ Your education or income level
- ☐ A physical disability
- ☐ Your shade of skin color
- ☐ Your tribe
- ☐ Other

Other, please specify: ______________________________

People who experience violence by their partner can often feel like they are going 'crazy' or 'losing their mind' or just lose hope and feel down all the time. Sometimes their partners may cause these feelings deliberately, or use these feelings as a way of trying to control them. In order to understand how this affects women, we'd like to ask you a few questions about some things that may have happened in your relationship.

Has your partner or ex-partner ever threatened to report to authorities that you are "crazy" to keep you from getting something you want or need (e.g., custody of children, medication, protective order)?

- ◯ Yes
- ◯ No

Do you think your partner or ex-partner has ever deliberately done things to make you feel like you are going crazy or losing your mind?	◯ Yes<br>◯ No
In the last few years, have you ever gone to see someone like a counselor or social worker or therapist or doctor to get help with feeling upset or depressed?	◯ Yes<br>◯ No
Has your partner or ex-partner ever tried to prevent or discourage you from getting that help or taking medication you were prescribed for your feelings?	◯ Yes<br>◯ No

The next questions address beliefs about equality in health care. Please indicate how much you personally disagree or agree with each statement.
(RESPONSE CARD PAGE 10)

	Strongly Disagree	Disagree	Neither Agree nor Disagree	Agree	Strongly Agree
Doctors and health care workers sometimes hide information from patients who belong to my race or ethnic group.	○	○	○	○	○
Doctors have the best interests of people of my race or ethnic group in mind.	○	○	○	○	○
People of my race or ethnic group should not confide in doctors and health care workers because it will be used against them.	○	○	○	○	○
People of my race or ethnic group should be suspicious of information from doctors and health care workers.	○	○	○	○	○
People of my race or ethnic group cannot trust doctors and health care workers.	○	○	○	○	○
People of my race or ethnic group should be suspicious of modern medicine.	○	○	○	○	○
Doctors and health care workers treat people of my race or ethnic group like "guinea pigs."	○	○	○	○	○
People of my race or ethnic group receive the same medical care from doctors and health care workers as people of other groups.	○	○	○	○	○
Doctors and health care workers do not take medical complaints of people in my race or ethnic group seriously.	○	○	○	○	○
People of my race or ethnic group are treated the same as people of other groups by doctors and health care workers.	○	○	○	○	○

In most hospitals, people of different race or ethnic groups receive the same kind of care.	○	○	○	○	○
I have personally been treated poorly or unfairly by doctors or health care workers because of my race or ethnicity.	○	○	○	○	○

Now I am going to ask you some questions about your opinion of law enforcement. Please tell me how strongly you agree or disagree with the following statements.
(RESPONSE CARD PAGE 11)

	Strongly Disagree	Disagree	Neither Agree Nor Disagree	Agree	Strongly Agree
Law enforcement officers sometimes hide information from people who belong to my racial group.	○	○	○	○	○
Law enforcement officers have the best interests of people of my racial group in mind.	○	○	○	○	○
People of my racial group should not confide in law enforcement officers because it will be used against them.	○	○	○	○	○
People of my racial group should be suspicious of information from law enforcement officers.	○	○	○	○	○
People of my racial group cannot trust law enforcement officers.	○	○	○	○	○
People of my racial group should be suspicious of the criminal justice system.	○	○	○	○	○
Law enforcement officers treat people of my racial group like "animals."	○	○	○	○	○
People of my racial group receive the same protection from law enforcement officers as people from other groups.	○	○	○	○	○
Law enforcement officers do not take the complaints of people of my racial group seriously.	○	○	○	○	○
People of my racial group are treated the same as people of other groups by law enforcement officers.	○	○	○	○	○
In most courts, people of different racial groups receive the same kind of treatment from the judge.	○	○	○	○	○

I have personally been treated poorly or unfairly by law enforcement officers because of my race.	◯	◯	◯	◯	◯

These next questions are about your current medical state and medical history.
Are you currently having any symptoms relating to your....

	Yes	No
Head, eyes, ears, nose, or throat	◯	◯
Breathing	◯	◯
Heart	◯	◯
Chest	◯	◯
Abdomen	◯	◯
Limbs	◯	◯
Skin	◯	◯
Other	◯	◯

Please explain: ______________________________

Have you ever been diagnosed with...

	Yes	No
A respiratory condition? (asthma, COPD, TB)	◯	◯
A heart or blood vessel condition? (heart disease, heart attack, stroke, varicose veins, blood clotting disorder)	◯	◯
An endocrine disorder? (diabetes, hyper or hypothyroidism)	◯	◯
Cancer?	◯	◯
A mental illness? (severe depression, schizophrenia, bipolar disorder)	◯	◯
An immune disorder? (lupus, Crohn's)	◯	◯
A gastrointestinal or liver condition?	◯	◯
A urinary or kidney condition?	◯	◯
A reproductive condition?	◯	◯
A muscular or skeletal disorder?	◯	◯
Another condition?	◯	◯

Please explain, including specific diagnosis, date of diagnosis, and any medications: ________________________________

In the past 2 weeks, have you used any antibiotics?
◯ Yes
◯ No

Within the past 30 days, have you taken PEP (Post-Exposure Prophylaxis) for HIV prevention?
◯ Yes
◯ No

In the past 30 days, have you used any of the following medicines or substances?

	Yes	No
Vaginal lubricant	○	○
Douches or vaginal washes	○	○
Antidepressants	○	○
Antipsychotics	○	○
Narcotic pain relievers (ex. Percocet, OxyContin)	○	○
Anti-anxiety medications	○	○
Steroid-based medications or corticosteroids	○	○
Medication for multiple sclerosis	○	○
Anti-fungals	○	○
Anti-diarrheals	○	○
Decongestants	○	○
Antidiuretics	○	○
Medication for Parkinson's	○	○
Medication for cholesterol reduction	○	○
Medication for anti-aging	○	○
Other medications prescribed by a doctor	○	○
Other over-the-counter medications	○	○

Please explain, including last date of use.

____

Section 5: Gynecologic and Reproductive History

SAY: These next questions are about your reproductive history.

At what age was your first period? ______________________

What was the date of the first day of your last period? ______________________

How many times have you been pregnant in your life, including abortions, miscarriages, and pregnancies that did not result in a live birth? ______________________

How many of these pregnancies ended in miscarriage? ______________________

How many of these pregnancies ended in stillbirth? ______________________

How many of these pregnancies ended in abortion? ______________________

How many of these pregnancies ended in ectopic or tubal pregnancy? ______________________

How many of these pregnancies ended in live birth by Cesarean section? ______________________

How many of these pregnancies ended in live birth by vaginal delivery? ______________________

Have you been pregnant in the last 12 months?
- ◯ Yes
- ◯ No

How many times have you been pregnant when you did not want to be? ______________________

How many of these pregnancies ended in miscarriage? ______________________

How many of these pregnancies ended in stillbirth? ______________________

How many of these pregnancies ended in abortion? ______________________

How many of these pregnancies ended in ectopic or tubal pregnancy? ______________________

How many of these pregnancies ended in live birth by Cesarean section? ______________________

How many of these pregnancies ended in live birth by vaginal delivery? ______________________

Have you ever had a complication during pregnancy (not labor or delivery)? Check all that apply	☐ Severe fatigue<br>☐ Severe abdominal pain (pain in the belly)<br>☐ Bleeding from the vagina<br>☐ Fever<br>☐ High blood sugar (diabetes or gestational diabetes)<br>☐ Heart Problems<br>☐ Unusual swelling of face, fingers, or legs<br>☐ Severe and continued headache<br>☐ Rapid breathing or difficult breathing<br>☐ Foul smelling vaginal discharge<br>☐ Convulsions or fits<br>☐ Loss of consciousness<br>☐ Blurred vision<br>☐ Placental abruption<br>☐ Other<br>☐ None<br>☐ Don't want to answer
Other, please specify:	______________________________
A preterm delivery is one that occurs at 36 weeks or earlier in pregnancy. As far as you know, have you ever had any preterm deliveries?	◯ Yes<br>◯ No
Were there any complications during labor and delivery for any of your pregnancies? (check all that apply)	☐ Heavy bleeding<br>☐ Labor pains lasting longer than 12 hours<br>☐ Vaginal tearing<br>☐ Convulsions<br>☐ Fever<br>☐ Green or brown water coming from the vagina<br>☐ Water breaks and labor is not induced within 6 hours<br>☐ Placenta not expelled within 1 hour of the birth<br>☐ Other<br>☐ None<br>☐ Don't want to answer
Other, please specify:	______________________________

What type of birth control methods have you EVER used? (check all that apply)	☐ Birth Control Pills (Progestin only)<br>☐ Birth Control Pills (Progestin and estrogen)<br>☐ Depo-Provera or injectables (or shots)<br>☐ Lunelle, a once a month injection<br>☐ Withdrawal or "pulling out'<br>☐ Rhythm or safe period by calendar<br>☐ Natural family planning or safe period by temperature or cervical mucus test<br>☐ Contraceptive patch<br>☐ Condom<br>☐ Vaginal ring (or "NuvaRing")<br>☐ "Morning after" pills or emergency contraception (Plan B)<br>☐ Male partner vasectomy or sterilization<br>☐ IUD<br>☐ Other<br>☐ None<br>☐ Don't want to answer<br>☐ Implant
Other, please specify:	______________________
What type of birth control methods have you used in the past 12 months? (check all that apply)	☐ Birth Control Pills (Progestin only)<br>☐ Birth Control Pills (Progestin and estrogen)<br>☐ Depo-Provera or injectables (or shots)<br>☐ Lunelle, a once a month injection<br>☐ Withdrawal or "pulling out'<br>☐ Rhythm or safe period by calendar<br>☐ Natural family planning or safe period by temperature or cervical mucus test<br>☐ Contraceptive patch<br>☐ Condom<br>☐ Vaginal ring (or "NuvaRing")<br>☐ "Morning after" pills or emergency contraception (Plan B)<br>☐ Male partner vasectomy or sterilization<br>☐ IUD<br>☐ Other<br>☐ None<br>☐ Don't want to answer<br>☐ Implant
Other, please specify:	______________________

What type of birth control methods do you use currently? (check all that apply)	☐ Birth Control Pills (Progestin only)<br>☐ Birth Control Pills (Progestin and estrogen)<br>☐ Depo-Provera or injectables (or shots)<br>☐ Lunelle, a once a month injection<br>☐ Withdrawal or "pulling out'<br>☐ Rhythm or safe period by calendar<br>☐ Natural family planning or safe period by temperature or cervical mucus test<br>☐ Contraceptive patch<br>☐ Condom<br>☐ Vaginal ring (or "NuvaRing")<br>☐ "Morning after" pills or emergency contraception (Plan B)<br>☐ Male partner vasectomy or sterilization<br>☐ IUD<br>☐ Other<br>☐ None<br>☐ Don't want to answer<br>☐ Implant
Other, please specify:	__________________________
How long have you used this method for? (in months)	__________________________
Before participating in this study, had you ever been tested for HIV?	◯ Had been tested<br>◯ Never tested<br>◯ Don't want to answer<br>◯ Don't remember
Before participating in this study, when was the last time you were tested for HIV?	__________________________
Before participating in this study, how many times had you been tested for HIV?	__________________________
Before participating in this study, had you ever had an HIV test result that you were not sure if the result was positive or negative?	◯ Yes<br>◯ No<br>◯ Don't know<br>◯ Don't want to answer<br>◯ Don't remember
How many times have you been tested for other sexually transmitted diseases like chlamydia, gonorrhea, or trichomoniasis?	__________________________

In your lifetime, have you EVER been told by a doctor that you had (check all that apply):

	Yes	No
Chlamydia	◯	◯
Gonorrhea	◯	◯
Trichomoniasis (Trich)	◯	◯
Syphilis	◯	◯
Genital Warts	◯	◯
Genital Herpes	◯	◯
Hepatitis B	◯	◯
Hepatitis C	◯	◯
HPV	◯	◯
Other	◯	◯

Other STI, please specify: ______________________

In the past six months, have you been told by a doctor that you had (check all that apply):

	Yes	No
Chlamydia	○	○
Gonorrhea	○	○
Trichomoniasis (Trich)	○	○
Syphilis	○	○
Genital Warts	○	○
Genital Herpes	○	○
Hepatitis B	○	○
Hepatitis C	○	○
HPV	○	○
Other	○	○

Other STI, please specify: ________________________________

Chlamydia: How many occurrences or outbreaks in the past 6 months? ________________________________

Gonorrhea: How many occurrences or outbreaks in the past 6 months? ________________________________

Trichomoniasis: How many occurrences or outbreaks in the past 6 months? ________________________________

Syphilis: How many occurrences or outbreaks in the past 6 months? ________________________________

Genital Warts: How many occurrences or outbreaks in the past 6 months? ________________________________

Genital Herpes: How many occurrences or outbreaks in the past 6 months? ________________________________

Hepatitis B: How many occurrences or outbreaks in the past 6 months? ________________________________

Hepatitis C: How many occurrences or outbreaks in the past 6 months? ________________________________

HPV: How many occurrences or outbreaks in the past 6 months? ________________________________

Other: How many occurrences or outbreaks in the past 6 months? ________________________________

In the past month, have you been told by a doctor that you had (check all that apply):

	Yes	No
Chlamydia	◯	◯
Gonorrhea	◯	◯
Trichomaniasis	◯	◯
Syphilis	◯	◯
Genital Warts	◯	◯
Genital Herpes	◯	◯
Hepatitis B	◯	◯
Hepatitis C	◯	◯
HPV	◯	◯
Other	◯	◯

Other STI, please specify: ______________________

Did you ever receive treatment for this/these?

- ◯ Yes, all of them
- ◯ Yes, some of them
- ◯ No, none of them
- ◯ Don't know
- ◯ Don't want to answer
- ◯ Don't remember

Which did you receive treatment for?

- ☐ Chlamydia
- ☐ Gonorrhea
- ☐ Trichomoniasis (Trich)
- ☐ Syphilis
- ☐ Genital Warts
- ☐ Genital Herpes
- ☐ Hepatitis B
- ☐ Hepatitis C
- ☐ HPV
- ☐ Other

Have you ever had the following (check all that apply):

	Yes	No
Urinary Tract Infection (UTI, Bladder infection, cystitis)	○	○
Yeast infection	○	○
Bacterial Vaginosis (BV)	○	○
Abnormal pap smear	○	○
Abnormal vaginal discharge	○	○
Pelvic Pain	○	○

Urinary Tract Infection: How many occurrences or outbreaks in the past 6 months? ______________________

Urinary Tract Infection: How many occurrences or outbreaks in the past month? ______________________

Yeast Infection: How many occurrences or outbreaks in the past 6 months? ______________________

Yeast Infection: How many occurrences or outbreaks in the past month? ______________________

Bacterial Vaginosis: How many occurrences or outbreaks in the past 6 months? ______________________

Bacterial Vaginosis: How many occurrences or outbreaks in the past month? ______________________

Abnormal Pap Smear: How many occurrences or outbreaks in the past 6 months? ______________________

Abnormal Pap Smear: How many occurrences or outbreaks in the past month? ______________________

Abnormal Vaginal Discharge: How many occurrences or outbreaks in the past 6 months? ______________________

Abnormal Vaginal Discharge: How many occurrences or outbreaks in the past month? ______________________

Pelvic Pain: How many occurrences or outbreaks in the past 6 months? ______________________

Pelvic Pain: How many occurrences or outbreaks in the past month? ______________________

Did you ever receive treatment for these?

- ○ Yes, all of them
- ○ Yes, some of them
- ○ No, none of them
- ○ Don't know
- ○ Don't want to answer
- ○ Don't remember

For which did you receive treatment?	☐ Urinary Tract Infection (UTI)<br>☐ Yeast Infection<br>☐ Bacterial Vaginosis (BV)<br>☐ Abnormal Pap Smea<br>☐ Abnormal Vaginal Discharge<br>☐ Pelvic Pain
Have you ever had unprotected vaginal or anal sex when you had STI symptoms (like discharge or pelvic pain) before receiving treatment?	◯ Yes<br>◯ No

Section 6: Sexual Behavior History

SAY: These first questions ask about the first time you had sex. By sex, we mean vaginal, anal, or oral sex. Before we get into specifics, here are a few definitions of these terms.

Vaginal sex means when a man inserts his penis into a woman's vagina.
Anal sex means when a man puts his penis into your anus or butt.
Oral sex means when a man puts his tongue or mouth in or around your vagina OR when you put your mouth or tongue on or around his penis.

There are no right or wrong answers to these questions and all responses you provide are confidential.

Was the event last week your first vaginal sexual experience with another person?	◯ Yes<br>◯ No
In the past 30 days, how frequently did you have vaginal sex?	◯ Never<br>◯ 1 time per week<br>◯ 2-3 times per week<br>◯ 4-5 times per week<br>◯ 7-10 times per week<br>◯ More than 10 times per week<br>◯ Do not want to answer
When having vaginal sex in the past 30 days, approximately how often did you and your partner use a barrier method of protection?	◯ Never<br>◯ Rarely<br>◯ Sometimes<br>◯ Often<br>◯ Always
Was the event last week your first anal sexual experience with another person?	◯ Yes<br>◯ No
In the past 30 days, how frequently did you have anal sex?	◯ Never<br>◯ 1 time per week<br>◯ 2-3 times per week<br>◯ 4-5 times per week<br>◯ 7-10 times per week<br>◯ More than 10 times per week<br>◯ Do not want to answer
When having anal sex in the past 30 days, approximately how often did you and your partner use a barrier method of protection?	◯ Never<br>◯ Rarely<br>◯ Sometimes<br>◯ Often<br>◯ Always
Was the event last week your first oral sexual experience with another person?	◯ Yes<br>◯ No

In the past 30 days, how frequently did you have oral sex?	◯ Never<br>◯ 1 time per week<br>◯ 2-3 times per week<br>◯ 4-5 times per week<br>◯ 7-10 times per week<br>◯ More than 10 times per week<br>◯ Do not want to answer
When having oral sex (giving or receiving) in the past 30 days, approximately how often did you and your partner use a barrier method of protection?	◯ Never<br>◯ Rarely<br>◯ Sometimes<br>◯ Often<br>◯ Always
In the past 48 hours, have you had vaginal sex without a condom?	◯ Yes<br>◯ No
In the past 48 hours, have you used a condom during sex or otherwise? (Ex., with a sex toy)	◯ Yes<br>◯ No
What type of condom did you use?	________________________________
In the past 48 hours, have you used vaginal lubricants?	◯ Yes<br>◯ No
In the past 48 hours, have you used sex toys?	◯ Yes<br>◯ No
What type of sex toys did you use?	________________________________
In your lifetime, how many consensual male sex partners have you had?<br><br>If don't know or don't want to answer, enter 999.	________________________________
In your lifetime, how many consensual female sex partners have you had?<br><br>If don't know or don't want to answer, enter 999.	________________________________
Were any of these partners concurrent (were you having sex with more than one of them during the same period of time)?	◯ Yes<br>◯ No<br>◯ Do not want to answer<br>◯ Do not remember
In the past six (6) months, how many consensual male sex partners have you had?<br><br>If don't know or don't want to answer, enter 999.	________________________________
In the past six (6) months, how many consensual female sex partners have you had?<br><br>If don't know or don't want to answer, enter 999.	________________________________

Were any of these partners concurrent (were you having sex with more than one of them during the same period of time)?

- ◯ Yes
- ◯ No
- ◯ Do not want to answer
- ◯ Do not remember

How old were you the first time you had vaginal sex with a man?

If don't know or don't want to answer, enter 999.

____

How would you describe this person?

- ◯ Spouse
- ◯ Steady or main partner
- ◯ Casual dating partner
- ◯ Friend
- ◯ Father
- ◯ Stepfather or my mother's boyfriend
- ◯ Other male family member
- ◯ Family Friend or Neighbor
- ◯ Stranger (someone you did not know)
- ◯ Other
- ◯ Don't want to answer

Other, please describe:

____

How old was this person?

If don't know or don't want to answer, enter 999.

____

Was this person at least 5 years older than you?

- ◯ Yes
- ◯ No
- ◯ Don't know
- ◯ Don't want to answer

How would you describe your first vaginal sex experience?

- ◯ Wanted and not forced
- ◯ Wanted but pressured
- ◯ Unwanted and pressured
- ◯ Unwanted and threatened with violence
- ◯ Unwanted and physically forced (hit, held down, slapped)
- ◯ Unwanted and forced to drink alcohol or take drugs
- ◯ Don't want to answer

Was a condom used during this encounter?

- ◯ Yes
- ◯ No
- ◯ I do not remember
- ◯ Don't want to answer

Did this person use alcohol or drugs immediately before or during the encounter?

- ◯ Yes
- ◯ No
- ◯ Don't know
- ◯ Don't want to answer

Did you use alcohol or drugs immediately before or during the encounter?

- ◯ Yes
- ◯ No
- ◯ Don't know
- ◯ Don't want to answer

How old were you the first time you had anal sex with a man?<br><br>If don't know or don't want to answer, enter 999.<br><br>If never had anal sex, enter 888.	__________________________
How would you describe this person?	◯ Spouse<br>◯ Steady or main partner<br>◯ Casual dating partner<br>◯ Friend<br>◯ Father<br>◯ Stepfather or my mother's boyfriend<br>◯ Other male family member<br>◯ Family Friend or Neighbor<br>◯ Stranger (someone you did not know)<br>◯ Other<br>◯ Don't want to answer
How old was this person?<br><br>If don't know or don't want to answer, enter 999.	__________________________
Was this person at least 5 years older than you?	◯ Yes<br>◯ No<br>◯ Don't know<br>◯ Don't want to answer
How would you describe your first anal sex experience?	◯ Wanted and not forced<br>◯ Wanted but pressured<br>◯ Unwanted and pressured<br>◯ Unwanted and threatened with violence<br>◯ Unwanted and physically forced (hit, held down, slapped)<br>◯ Unwanted and forced to drink alcohol or take drugs<br>◯ Don't want to answer
Was a condom used during this encounter?	◯ Yes<br>◯ No<br>◯ I do not remember<br>◯ Don't want to answer
Did this person use alcohol or drugs immediately before or during the encounter?	◯ Yes<br>◯ No<br>◯ Don't know<br>◯ Don't want to answer
Did you use alcohol or drugs immediately before or during the encounter?	◯ Yes<br>◯ No<br>◯ Don't know<br>◯ Don't want to answer
How old were you the first time you had oral sex with a man?<br><br>If don't know or don't want to answer, enter 999.<br><br>If never had oral sex, enter 888.	__________________________

How would you describe this person?	◯ Spouse<br>◯ Steady or main partner<br>◯ Casual dating partner<br>◯ Friend<br>◯ Father<br>◯ Stepfather or my mother's boyfriend<br>◯ Other male family member<br>◯ Family Friend or Neighbor<br>◯ Stranger (someone you did not know)<br>◯ Other<br>◯ Don't want to answer
How old was this person?<br><br>If don't know or don't want to answer, enter 999.	__________________________
Was this person at least 5 years older than you?	◯ Yes<br>◯ No<br>◯ Don't know<br>◯ Don't want to answer
How would you describe your first oral sex experience?	◯ Wanted and not forced<br>◯ Wanted but pressured<br>◯ Unwanted and pressured<br>◯ Unwanted and threatened with violence<br>◯ Unwanted and physically forced (hit, held down, slapped)<br>◯ Unwanted and forced to drink alcohol or take drugs<br>◯ Don't want to answer
Was a condom used during this encounter?	◯ Yes<br>◯ No<br>◯ I do not remember<br>◯ Don't want to answer
Did this person use alcohol or drugs immediately before or during the encounter?	◯ Yes<br>◯ No<br>◯ Don't know<br>◯ Don't want to answer
Did you use alcohol or drugs immediately before or during the encounter?	◯ Yes<br>◯ No<br>◯ Don't know<br>◯ Don't want to answer
(Other than last week) Has a male sex partner ever used threats to make you have sex when you did not want to? (If yes, how many times?)	◯ Never<br>◯ 1 time<br>◯ 2-3 times<br>◯ 4-5 times<br>◯ 6-7 times<br>◯ 8-9 times<br>◯ More than 10 times<br>◯ Don't Know<br>◯ Don't want to answer

(Other than last week) Has a male sex partner ever used force (like hitting, holding down, or using a weapon) to make you have sex with him?	◯ Never<br>◯ 1 time<br>◯ 2-3 times<br>◯ 4-5 times<br>◯ 6-7 times<br>◯ 8-9 times<br>◯ More than 10 times<br>◯ Don't Know<br>◯ Don't want to answer
(Other than last week) Has any other male ever used threats to make you have sex when you did not want to?	◯ Never<br>◯ 1 time<br>◯ 2-3 times<br>◯ 4-5 times<br>◯ 6-7 times<br>◯ 8-9 times<br>◯ More than 10 times<br>◯ Don't Know<br>◯ Don't want to answer
(Other than last week) Has any other male ever used force (like hitting, holding down, or using a weapon) to make you have sex with her?	◯ Never<br>◯ 1 time<br>◯ 2-3 times<br>◯ 4-5 times<br>◯ 6-7 times<br>◯ 8-9 times<br>◯ More than 10 times<br>◯ Don't Know<br>◯ Don't want to answer
Has a female sex partner ever used threats to make you have sex when you did not want to?	◯ Never<br>◯ 1 time<br>◯ 2-3 times<br>◯ 4-5 times<br>◯ 6-7 times<br>◯ 8-9 times<br>◯ More than 10 times<br>◯ Don't Know<br>◯ Don't want to answer
Has a female sex partner ever used force (like hitting, holding down, or using a weapon) to make you have sex with her?	◯ Never<br>◯ 1 time<br>◯ 2-3 times<br>◯ 4-5 times<br>◯ 6-7 times<br>◯ 8-9 times<br>◯ More than 10 times<br>◯ Don't Know<br>◯ Don't want to answer
Has any other female ever used threats to make you have sex when you did not want to?	◯ Never<br>◯ 1 time<br>◯ 2-3 times<br>◯ 4-5 times<br>◯ 6-7 times<br>◯ 8-9 times<br>◯ More than 10 times<br>◯ Don't Know<br>◯ Don't want to answer

Has any other female ever used force (like hitting, holding down, or using a weapon) to make you have sex with her?	◯ Never<br>◯ 1 time<br>◯ 2-3 times<br>◯ 4-5 times<br>◯ 6-7 times<br>◯ 8-9 times<br>◯ More than 10 times<br>◯ Don't Know<br>◯ Don't want to answer

Section 7: Substance Use History

The next set of questions ask about your use of tobacco, alcohol, and other substances.

How old were you when you first used a tobacco product? (Ex. Cigarette, e-cigarette cigar, little cigar/cigarillo, chew, etc)

If never used tobacco, enter 888.
If do not want to answer, enter 999.

____

How old were you were you first drank alcohol?

If never used alcohol, enter 888.
If do not want to answer, enter 999.

____

How old were you when you first used drugs (including cannabis, prescription drugs for non-prescription use, and illegal substances?

If never used drugs, enter 888.
If do not want to answer, enter 999.

____

What specific drug(s) did you use (the first time)?

____

Thinking about the past 6 months, how often did you use alcohol immediately before or during sex? (RESPONSE CARD PAGE 12)

- ◯ Never
- ◯ Once
- ◯ 2-3 times
- ◯ 4-6 times
- ◯ 7 or more times
- ◯ Do not want to answer

In your lifetime, how often have you used alcohol immediately before or during sex?

- ◯ Never
- ◯ Once
- ◯ 2-3 times
- ◯ 4-6 times
- ◯ 7 or more times
- ◯ Do not want to answer

Thinking about the past 6 months, how often did you use drugs (including cannabis, prescription drugs, and illegal substances) immediately before or during sex?

- ◯ Never
- ◯ Once
- ◯ 2-3 times
- ◯ 4-6 times
- ◯ 7 or more times
- ◯ Do not want to answer

In your lifetime, how often have you used drugs (including cannabis, prescription drugs, and illegal substances) immediately before or during sex?

- ◯ Never
- ◯ Once
- ◯ 2-3 times
- ◯ 4-6 times
- ◯ 7 or more times
- ◯ Do not want to answer

In the past year, how often have you used the following?
(RESPONSE CARD PAGE 13)

	Never	Once or Twice	Monthly	Weekly	Daily or Almost Daily
Alcohol	◯	◯	◯	◯	◯
Tobacco Products	◯	◯	◯	◯	◯
Prescriptions drugs for Non-Medical Reasons	◯	◯	◯	◯	◯
Other Drugs (including marijuana)	◯	◯	◯	◯	◯

How often do you have a drink of alcohol? (RESPONSE CARD PAGE 14)	◯ Never<br>◯ Monthly or less<br>◯ 2-4 times per month<br>◯ 2-3 times per week<br>◯ 4 or more times a week
How many standard drinks containing alcohol do you have on a typical day?	◯ 1 or 2<br>◯ 3 or 4<br>◯ 5 or 6<br>◯ 7 to 9<br>◯ 10 or more
How often do you have 6 or more drinks on one occasion? (RESPONSE CARD PAGE 15)	◯ Never<br>◯ Less than monthly<br>◯ Monthly<br>◯ Weekly<br>◯ Daily or almost daily
How often during the last year have you found that you were not able to stop drinking on you had started? (RESPONSE CARD PAGE 16)	◯ Never<br>◯ Monthly or less<br>◯ 2-4 times per month<br>◯ 2-3 times per week<br>◯ 4 or more times a week
How often during the last year have you failed to do what was normally expected from you because of drinking?	◯ Never<br>◯ Monthly or less<br>◯ 2-4 times per month<br>◯ 2-3 times per week<br>◯ 4 or more times a week
How often during the past year have you needed a first drink in the morning to get yourself going after a heavy drinking session?	◯ Never<br>◯ Monthly or less<br>◯ 2-4 times per month<br>◯ 2-3 times per week<br>◯ 4 or more times a week
How often during the last year have you had a feeling of guilt or remorse after drinking?	◯ Never<br>◯ Monthly or less<br>◯ 2-4 times per month<br>◯ 2-3 times per week<br>◯ 4 or more times a week

How often during the last year have you been unable to remember what happened the night before because you had been drinking?	◯ Never<br>◯ Monthly or less<br>◯ 2-4 times per month<br>◯ 2-3 times per week<br>◯ 4 or more times a week
Have you or someone else been injured as a result of your drinking?	◯ No<br>◯ Yes, but not during the last year<br>◯ Yes, during the last year
Has a relative or a doctor or another health worker been concerned about your drinking or suggested you cut down?	◯ No<br>◯ Yes, but not during the last year<br>◯ Yes, during the last year

In your lifetime, which of the of the following substances have you ever used?

	Yes	No
Cannabis (marijuana, pot, grass, hash, etc.)	○	○
Cocaine (coke, crack, etc.)	○	○
Prescriptions Stimulants (Ritalin, Concerta, Dexedrine, Adderall, diet pills, etc.)	○	○
Methamphetamine (speed, cystal meth, Ecstasy, etc.)	○	○
Inhalants (Nitrous oxide, glue, gas, paint thinner, etc.)	○	○
Sedatives or sleeping pills (Valium, Serepax, Ativan, Xanax, Liberium, Rohypnol, GHB, etc.)	○	○
Hallucinogens (LSD, acid, mushrooms, PCP, Special K, etc.)	○	○
Street opioids (heroin, opium, etc.)	○	○
Perscription opioids (Fentanyl, oxycodone [OxyCotin, Percocet], hydrocodone [Vicodin], methodone, buprenorphine, Codeine, morphine, etc.)	○	○
Other	○	○

Other, please specify: ______________________

In the past three months, how often have you used the substances you mentioned?

	Never	Once or twice	Monthly	Weekly	Daily or almost daily
Cannabis (marijuana, pot, grass, hash, etc.)	○	○	○	○	○
Cocaine (coke, crack, etc.)	○	○	○	○	○
Prescriptions Stimulants (Ritalin, Concerta, Dexedrine, Adderall, diet pills, etc.)	○	○	○	○	○
Methamphetamine (speed, crystal meth, Ecstasy, etc.)	○	○	○	○	○
Inhalants (Nitrous oxide, glue, gas, paint thinner, etc.)	○	○	○	○	○
Sedatives or sleeping pills (Valium, Serepax, Ativan, Xanax, Liberium, Rohypnol, GHB, etc.)	○	○	○	○	○
Hallucinogens (LSD, acid, mushrooms, PCP, Special K, Ecstasy, etc.)	○	○	○	○	○
Street opioids (heroin, opium, etc.)	○	○	○	○	○
Perscription opioids (Fentanyl, oxycodone [OxyCotin, Percocet], hydrocodone [Vicodin], methodone, buprenorphine, Codeine, morphine, etc.)	○	○	○	○	○
Other, as previously specified	○	○	○	○	○

In the past three months, how often have you had a strong desire or urge to use cannabis? (marijuana, pot, grass, hash, etc.)
(RESPONSE CARD PAGE 17)

- ○ Never
- ○ Once or twice
- ○ Monthly
- ○ Weekly
- ○ Daily or almost daily

In the past three months, how often have you had a strong desire or urge to use cocaine? (coke, crack, etc.)

- ○ Never
- ○ Once or twice
- ○ Monthly
- ○ Weekly
- ○ Daily or almost daily

In the past three months, how often have you had a strong desire or urge to use prescriptions stimulants? (Ritalin, Concerta, Dexedrine, Adderall, diet pills, etc.)

- ○ Never
- ○ Once or twice
- ○ Monthly
- ○ Weekly
- ○ Daily or almost daily

In the past three months, how often have you had a strong desire or urge to use methamphetamine? (speed, crystal meth, Ecstasy, etc.)	◯ Never<br>◯ Once or twice<br>◯ Monthly<br>◯ Weekly<br>◯ Daily or almost daily
In the past three months, how often have you had a strong desire or urge to use inhalants? (Nitrous oxide, glue, gas, paint thinner, etc.)	◯ Never<br>◯ Once or twice<br>◯ Monthly<br>◯ Weekly<br>◯ Daily or almost daily
In the past three months, how often have you had a strong desire or urge to use sedatives or sleeping pills? (Valium, Serepax, Ativan, Xanax, Liberium, Rohypnol, GHB, etc.)	◯ Never<br>◯ Once or twice<br>◯ Monthly<br>◯ Weekly<br>◯ Daily or almost daily
In the past three months, how often have you had a strong desire or urge to use hallucinogens? (LSD, acid, mushrooms, PCP, Special K, Ecstasy, etc.)	◯ Never<br>◯ Once or twice<br>◯ Monthly<br>◯ Weekly<br>◯ Daily or almost daily
In the past three months, how often have you had a strong desire or urge to use street opioids? (heroin, opium, etc.)	◯ Never<br>◯ Once or twice<br>◯ Monthly<br>◯ Weekly<br>◯ Daily or almost daily
In the past three months, how often have you had a strong desire or urge to use prescription opioids? (Fentanyl, oxycodone [OxyCotin, Percocet], hydrocodone [Vicodin], methodone, buprenorphine, Codeine, morphine, etc.)	◯ Never<br>◯ Once or twice<br>◯ Monthly<br>◯ Weekly<br>◯ Daily or almost daily
In the past three months, how often have you had a strong desire or urge to use other drugs, as previously specified?	◯ Never<br>◯ Once or twice<br>◯ Monthly<br>◯ Weekly<br>◯ Daily or almost daily
During the past three months, how often has your use of cannabis (marijuana, pot, grass, hash, etc.) led to health, social, legal or financial problems?	◯ Never<br>◯ Once or twice<br>◯ Monthly<br>◯ Weekly<br>◯ Daily or almost daily
During the past three months, how often has your use of cocaine (coke, crack, etc.) led to health, social, legal or financial problems?	◯ Never<br>◯ Once or twice<br>◯ Monthly<br>◯ Weekly<br>◯ Daily or almost daily
During the past three months, how often has your use of prescriptions stimulants (Ritalin, Concerta, Dexedrine, Adderall, diet pills, etc.) led to health, social, legal or financial problems?	◯ Never<br>◯ Once or twice<br>◯ Monthly<br>◯ Weekly<br>◯ Daily or almost daily

During the past three months, how often has your use of methamphetamine (speed, crystal meth, Ecstasy, etc.) led to health, social, legal or financial problems?	◯ Never<br>◯ Once or twice<br>◯ Monthly<br>◯ Weekly<br>◯ Daily or almost daily
During the past three months, how often has your use of inhalants (Nitrous oxide, glue, gas, paint thinner, etc.) led to health, social, legal or financial problems?	◯ Never<br>◯ Once or twice<br>◯ Monthly<br>◯ Weekly<br>◯ Daily or almost daily
During the past three months, how often has your use of sedatives or sleeping pills(Valium, Serepax, Ativan, Xanax, Liberium, Rohypnol, GHB, etc.) led to health, social, legal or financial problems?	◯ Never<br>◯ Once or twice<br>◯ Monthly<br>◯ Weekly<br>◯ Daily or almost daily
During the past three months, how often has your use of hallucinogens (LSD, acid, mushrooms, PCP, Special K, Ecstasy, etc.) led to health, social, legal or financial problems?	◯ Never<br>◯ Once or twice<br>◯ Monthly<br>◯ Weekly<br>◯ Daily or almost daily
During the past three months, how often has your use of street opioids (heroin, opium, etc.) led to health, social, legal or financial problems?	◯ Never<br>◯ Once or twice<br>◯ Monthly<br>◯ Weekly<br>◯ Daily or almost daily
During the past three months, how often has your use of prescription opioids (Fentanyl, oxycodone [OxyCotin, Percocet], hydrocodone [Vicodin], methodone, buprenorphine, Codeine, morphine, etc.) led to health, social, legal or financial problems?	◯ Never<br>◯ Once or twice<br>◯ Monthly<br>◯ Weekly<br>◯ Daily or almost daily
During the past three months, how often has your use of other drugs, as previously specified, led to health, social, legal or financial problems?	◯ Never<br>◯ Once or twice<br>◯ Monthly<br>◯ Weekly<br>◯ Daily or almost daily
During the past three months, how often have you failed to do what was normally expected of you because of your use of cannabis? (marijuana, pot, grass, hash, etc.)	◯ Never<br>◯ Once or twice<br>◯ Monthly<br>◯ Weekly<br>◯ Daily or almost daily
During the past three months, how often have you failed to do what was normally expected of you because of your use of cocaine? (coke, crack, etc.)	◯ Never<br>◯ Once or twice<br>◯ Monthly<br>◯ Weekly<br>◯ Daily or almost daily
During the past three months, how often have you failed to do what was normally expected of you because of your use of prescriptions stimulants? (Ritalin, Concerta, Dexedrine, Adderall, diet pills, etc.)	◯ Never<br>◯ Once or twice<br>◯ Monthly<br>◯ Weekly<br>◯ Daily or almost daily

During the past three months, how often have you failed to do what was normally expected of you because of your use of methamphetamine? (speed, crystal meth, Ecstasy, etc.)	◯ Never<br>◯ Once or twice<br>◯ Monthly<br>◯ Weekly<br>◯ Daily or almost daily
During the past three months, how often have you failed to do what was normally expected of you because of your use of inhalants? (Nitrous oxide, glue, gas, paint thinner, etc.)	◯ Never<br>◯ Once or twice<br>◯ Monthly<br>◯ Weekly<br>◯ Daily or almost daily
During the past three months, how often have you failed to do what was normally expected of you because of your use of sedatives or sleeping pills? (Valium, Serepax, Ativan, Xanax, Liberium, Rohypnol, GHB, etc.)	◯ Never<br>◯ Once or twice<br>◯ Monthly<br>◯ Weekly<br>◯ Daily or almost daily
During the past three months, how often have you failed to do what was normally expected of you because of your use of hallucinogens? (LSD, acid, mushrooms, PCP, Special K, Ecstasy, etc.)	◯ Never<br>◯ Once or twice<br>◯ Monthly<br>◯ Weekly<br>◯ Daily or almost daily
During the past three months, how often have you failed to do what was normally expected of you because of your use of street opioids? (heroin, opium, etc.)	◯ Never<br>◯ Once or twice<br>◯ Monthly<br>◯ Weekly<br>◯ Daily or almost daily
During the past three months, how often have you failed to do what was normally expected of you because of your use of prescription opioids? (Fentanyl, oxycodone [OxyCotin, Percocet], hydrocodone [Vicodin], methodone, buprenorphine, Codeine, morphine, etc.)	◯ Never<br>◯ Once or twice<br>◯ Monthly<br>◯ Weekly<br>◯ Daily or almost daily
During the past three months, how often have you failed to do what was normally expected of you because of your use of other drugs, as previously specified?	◯ Never<br>◯ Once or twice<br>◯ Monthly<br>◯ Weekly<br>◯ Daily or almost daily

Has a friend or relative or anyone else ever expressed concern about your use of the following drugs?

	No, Never	Yes, but not in the past 3 months	Yes, in the past 3 months
Cannabis (marijuana, pot, grass, hash, etc.)	○	○	○
Cocaine (coke, crack, etc.)	○	○	○
Prescriptions Stimulants (Ritalin, Concerta, Dexedrine, Adderall, diet pills, etc.)	○	○	○
Methamphetamine (speed, crystal meth, Ecstasy, etc.)	○	○	○
Inhalants (Nitrous oxide, glue, gas, paint thinner, etc.)	○	○	○
Sedatives or sleeping pills (Valium, Serepax, Ativan, Xanax, Liberium, Rohypnol, GHB, etc.)	○	○	○
Hallucinogens (LSD, acid, mushrooms, PCP, Special K, Ecstasy, etc.)	○	○	○
Street opioids (heroin, opium, etc.)	○	○	○
Prescription opioids (Fentanyl, oxycodone [OxyCotin, Percocet], hydrocodone [Vicodin], methodone, buprenorphine, Codeine, morphine, etc.)	○	○	○
Other, as previously specified.	○	○	○

Have you ever tried and failed to control, cut down or stop using the following drug(s)?

	No, Never	Yes, but not in the past 3 months	Yes, in the past 3 months
Cannabis (marijuana, pot, grass, hash, etc.)	◯	◯	◯
Cocaine (coke, crack, etc.)	◯	◯	◯
Prescriptions Stimulants (Ritalin, Concerta, Dexedrine, Adderall, diet pills, etc.)	◯	◯	◯
Methamphetamine (speed, cystal meth, Ecstasy, etc.)	◯	◯	◯
Inhalants (Nitrous oxide, glue, gas, paint thinner, etc.)	◯	◯	◯
Sedatives or sleeping pills (Valium, Serepax, Ativan, Xanax, Liberium, Rohypnol, GHB, etc.)	◯	◯	◯
Hallucinogens (LSD, acid, mushrooms, PCP, Special K, Ecstasy, etc.)	◯	◯	◯
Street opioids (heroin, opium, etc.)	◯	◯	◯
Prescription opioids (Fentanyl, oxycodone [OxyCotin, Percocet], hydrocodone [Vicodin], methodone, buprenorphine, Codeine, morphine, etc.)	◯	◯	◯
Other, as previously specified.	◯	◯	◯

Have you ever used any drug by injection, other than those prescribed by a doctor?	◯ No, never<br>◯ Yes, but not in the past 3 months<br>◯ Yes, in the past 3 months
Have you ever shared needles when injecting drugs?	◯ Yes<br>◯ No
Have you ever used any substances, including alcohol and drugs, in combination (at the same time)?	◯ No<br>◯ Yes, drugs and alcohol<br>◯ Yes, multiple kids of drugs<br>◯ Don't remember<br>◯ Don't want to answer

Which of the following methods have you used to do drugs?	☐ Injecting<br>☐ Snorting<br>☐ Sniffing<br>☐ Smoking<br>☐ Oral consumption (ex. food or pills)<br>☐ Huffing<br>☐ Other<br>☐ Don't want to answer<br>☐ Don't remember
Other, please specify:	__________________________________

Sometimes people are dealing with a partner who is trying to maintain power and control in the relationship. Sometimes, they may use substance abuse as a way to maintain that power and control. In order to understand how this affects women, we'd like to ask you a few questions about some things that may have happened in your relationship.

Has your partner or ex-partner ever pressured or forced you to use alcohol or other drugs or made you use more than you wanted?	◯ Yes<br>◯ No
Has your partner or ex-partner ever threatened to report your alcohol or other drug use to anyone in authority to keep you from getting something you want or need (e.g., custody of children, a job, benefits, or a protective order)?	◯ Yes<br>◯ No
Have you ever been afraid to call the police for help because your partner or ex-partner said they wouldn't believe you because you were using, or you would be arrested for being under the influence of alcohol or drugs?	◯ Yes<br>◯ No
In the last few years, have you ever tried to get help for your use of alcohol or other drugs?	◯ Yes<br>◯ No
Has your partner or ex-partner ever tried to prevent or discourage you from getting that help?	◯ Yes<br>◯ No

Section 8: Sexual Assault (Cases only- will not appear for controls)

The next set of questions are directly about your experience with sexual assault or non-consensual sex. Please know that you can pause or take a break at any time. Also, please remember that all responses are confidential.

Prior to the event, did the perpetrator use any drugs or alcohol?	◯ Yes<br>◯ No<br>◯ Don't know/Don't remember
Prior to the event, the perpetrator force you to take, or give you without your knowledge, any drugs or alcohol?	◯ Yes<br>◯ No<br>◯ Dont' know/Don't remember
Prior to the event had you used any drugs or alcohol voluntarily?	◯ Yes<br>◯ No<br>◯ Don't know/Don't remember
Following your experience with non-consensual sex last week, did you seek services or support anywhere?	◯ Yes<br>◯ No
Did you seek....	☐ Emotional Support<br>☐ Health-related services (for medical needs, injuries, emergency contraception, STI testing, etc.)<br>☐ Law Enforcement services?<br>☐ Legal services?<br>☐ Shelter or housing services?<br>☐ Other services or support?
Where or with whom did you seek emotional support? (Select all that apply)	☐ A friend<br>☐ A family member<br>☐ A religious member or religious services<br>☐ Counseling/Therapy services<br>☐ Group therapy/Support group (e.g. with peers and/or therapist)<br>☐ Crisis center (i.e., Center for Community Solutions)<br>☐ Hotline/helpline<br>☐ Mobile App<br>☐ Other
Other, please explain:	______________________________
Where did you seek health related services?	☐ Emergency Room<br>☐ Urgent care<br>☐ Crisis center (i.e., Center for Community Solutions)<br>☐ School health center<br>☐ Primary care doctor or other doctor<br>☐ Pharmacy<br>☐ Other
Other, please explain.	______________________________

Where else did you seek services or support?

____

Section 9: Post-Event Change (Cases only- will not appear for controls)

The next few questions ask about how you've been feeling over the past week.

During the past week, has your substance use changed?	◯ Yes<br>◯ No
How has your substance use changed in the past week?	◯ Increased use of alcohol<br>◯ Decreased use of alcohol<br>◯ Increased use of drugs<br>◯ Decreased use of drugs<br>◯ Other<br>◯ Don't want to answer<br>◯ Don't remember
Other, please specify:	__________________________________

For the next few questions, think about how you felt during the past week. During the past week, have you...

	Yes	No
Have had nightmares about it or thought about it when you did not want to?	◯	◯
Tried hard not to think about it or went out of your way to avoid situations that reminded you of it?	◯	◯
Were constantly on guard, watchful, or easily startled?	◯	◯
Felt numb or detached from others, activities, or your surroundings?	◯	◯

Thinking about the past week, indicate how often you felt the following ways. (RESPONSE CARD PAGE 1)

	Rarely or none of the time (less than 1 day per week)	Some or a little of the time (1-2 days per week)	Occasionally or a moderate amount of time (3-4 days per week)	All of the time (5-7 days per week)
I was bothered by things that usually don't bother me.	◯	◯	◯	◯
I had trouble keeping my mind on what I was going.	◯	◯	◯	◯
I felt depressed.	◯	◯	◯	◯
I felt that everything I did was an effort.	◯	◯	◯	◯
I felt hopeful about the future.	◯	◯	◯	◯
I felt fearful.	◯	◯	◯	◯
My sleep was restless.	◯	◯	◯	◯
I was happy.	◯	◯	◯	◯
I felt lonely.	◯	◯	◯	◯
I could not "get going."	◯	◯	◯	◯

Thinking about the past week, how often did you feel the following ways? (RESPONSE CARD PAGE 2)

	Never	Almost Never	Sometimes	Fairly Often	Very Often
How often have you been upset because of something that happened unexpectedly?	○	○	○	○	○
How often have you felt hat you were unable to control the important things in your life?	○	○	○	○	○
How often have you felt nervous and "stressed"?	○	○	○	○	○
How often have you felt confident about your ability to handle your personal problems?	○	○	○	○	○
How often have you felt that things were going your way?	○	○	○	○	○
How often have you found that you could not cope with all the things you had to do?	○	○	○	○	○
How often have you been able to control irritations in your life?	○	○	○	○	○
How often have you felt that you were on top of things?	○	○	○	○	○
How often have you been angered because of things that were outside of your control?	○	○	○	○	○
How often have you felt that difficulties were piling up so high that you could not overcome them?	○	○	○	○	○

Section 10: Referral Assessment

Have you ever thought about or attempted to kill yourself?	◯ Never<br>◯ It was just a brief passing thought<br>◯ I have had a plan to kill myself at least once to kill myself but did not try to do it<br>◯ I have had a plan to kill myself and really wanted to die<br>◯ I have attempted to kill myself, but did not want to die<br>◯ I have attempted to kill myself, and really hoped to die
SBQ1 Score	______________________
How often have you thought about killing yourself in the past year?	◯ Never<br>◯ Rarely (1 time)<br>◯ Sometimes (2 times)<br>◯ Often (3-4 times)<br>◯ Very Often (5 or more times)
SBQ2 Score	______________________
Have you ever told someone that you were going to commit suicide, or that you might do it?	◯ No<br>◯ Yes, at one time, but did not really want to die<br>◯ Yes, at one time, and really wanted to die<br>◯ Yes, more than once, but did not want to do it<br>◯ Yes, more than once, and really wanted to do it
SBQ3 Score	______________________
How likely is it that you will attempt suicide someday?<br>(RESPONSE CARD PAGE 18)	◯ Never<br>◯ No chance at all<br>◯ Rather unlikely<br>◯ Unlikely<br>◯ Likely<br>◯ Rather likely<br>◯ Very likely
SBQ4 Score	______________________
Suicidality Assessment Score: If score is 7 or above, conduct suicidality protocol.	______________________
Thinking about your most recent relationship, has your partner or ex-partner been very jealous and controlling, like keeping you from family and friends?	◯ Yes<br>◯ No
Thinking about your most recent relationship, has your partner or ex-partner ever been physically violent with you? (ex. Punching, kicking, slammed you against the wall, beat you up?)	◯ Yes<br>◯ No

Several risk factors have been associated with increased risk of homicides (murders) of women in violent relationships. We cannot predict what will happen in your case, but we want you to be aware of the danger of homicide in abusive relationships. These questions identify how many risk factors apply to your situation. At the end of this survey the data collector will discuss the results of this section with you.

	Yes	No
Has the physical violence increased in severity or frequency over the past year?	○	○
Does he own a gun?	○	○
Have you ever lived with him?	○	○
Have you left him after living together during the past year?	○	○
Is he unemployed?	○	○
Has he ever used a weapon against you or threatened you with a lethal weapon?	○	○
Was the weapon a gun?	○	○
Does he threaten to kill you?	○	○
Has he avoided being arrested for domestic violence?	○	○
Do you have a child that is not his?	○	○
Has he ever forced you to have sex when you did not wish to do so?	○	○
Does he ever try to choke you?	○	○
Does he use illegal drugs? By drugs, I mean "uppers" or amphetamines, meth, speed, angel dust, cocaine, "crack," street drugs or mixtures.	○	○
Is he an alcoholic or problem drinker?	○	○
Does he control most or all of your daily activities? (For instance: does he tell you who you can be friends with, when you can see your family, how much money you can use, or when you can take the car?)	○	○
Does he try to control you, but you do not let him?	○	○

Is he violently and constantly jealous of you? (For instance, does he say "If I can't have you, no one can.")	◯	◯
Have you ever been beaten by him while you were pregnant?	◯	◯
Have you ever been pregnant by him?	◯	◯
Has he ever threatened or tried to commit suicide?	◯	◯
Does he threaten to harm your children?	◯	◯
Do you believe he is capable of killing you?	◯	◯
Does he follow or spy on you, leave threatening notes or messages on answering machine, destroy your property, or call you when you don't want him to call?	◯	◯

Have you ever threatened or tried to commit suicide?
◯ Yes
◯ No

DA Score ______________________

DA Classification: ______________________

If score is 3 or 4, complete HIGH DANGER Protocol
If score is 1 or 2 complete UNSAFE RELATIONSHIP Protocol
If score is NULL (no value) complete COMMUNITY AWARENESS Protocol

This concludes the survey for today. Thank you very much for your time and honesty in completing this. We greatly appreciate both. Now we are going to move on to the exam portion of the study visit.

Notes on baseline data collection: ____________________________
